# Genetic and Transcription Profile Analysis of Tissue-Specific Anthocyanin Pigmentation in Carrot Root Phloem

**DOI:** 10.3390/genes12101464

**Published:** 2021-09-22

**Authors:** Florencia Bannoud, Sofia Carvajal, Shelby Ellison, Douglas Senalik, Sebastian Gomez Talquenca, Massimo Iorizzo, Philipp W. Simon, Pablo F. Cavagnaro

**Affiliations:** 1 Consejo Nacional de Investigaciones Científicas y Técnicas (CONICET), Av. Rivadavia 1917, Buenos Aires C1033, Argentina; fbannoud@mendoza-conicet.gob.ar (F.B.); carvajal.sofia@inta.gob.ar (S.C.); 2 Department of Horticulture, University of Wisconsin-Madison, 1575 Linden Drive, Madison, WI 53706, USA; slrepinski@wisc.edu; 3 USDA-Agricultural Research Service, Vegetable Crops Research Unit, University of Wisconsin-Madison, 1575 Linden Drive, Madison, WI 53706, USA; douglas.senalik@usda.gov; 4 Instituto Nacional de Tecnología Agropecuaria (INTA) EEA Mendoza, San Martin 3853, Luján de Cuyo, Mendoza M5507, Argentina; gomez.talquenca@inta.gob.ar; 5 Plants for Human Health Institute, North Carolina State University, Kannapolis, NC 28081, USA; miorizz@ncsu.edu; 6 Department of Horticultural Science, North Carolina State University, Raleigh, NC 27695, USA; 7 Instituto Nacional de Tecnología Agropecuaria (INTA) EEA La Consulta, Ex Ruta 40 km 96, La Consulta, San Carlos, Mendoza M5567, Argentina; 8 Instituto de Horticultura, Facultad de Ciencias Agrarias, Universidad Nacional de Cuyo, Luján de Cuyo, Mendoza M5528, Argentina

**Keywords:** *Daucus carota*, anthocyanin, phloem pigmentation, genetic mapping, RNA-Seq, candidate genes, RT-qPCR

## Abstract

In purple carrots, anthocyanin pigmentation can be expressed in the entire root, or it can display tissue specific-patterns. Within the phloem, purple pigmentation can be found in the outer phloem (OP) (also called the cortex) and inner phloem (IP), or it can be confined exclusively to the OP. In this work, the genetic control underlying tissue-specific anthocyanin pigmentation in the carrot root OP and IP tissues was investigated by means of linkage mapping and transcriptome (RNA-seq) and phylogenetic analyses; followed by gene expression (RT-qPCR) evaluations in two genetic backgrounds, an F_2_ population (3242) and the inbred B7262. Genetic mapping of ‘root outer phloem anthocyanin pigmentation’ (*ROPAP*) and inner phloem pigmentation (*RIPAP*) revealed colocalization of *ROPAP* with the *P*_1_ and *P*_3_ genomic regions previously known to condition pigmentation in different genetic stocks, whereas *RIPAP* co-localized with *P*_3_ only. Transcriptome analysis of purple OP (POP) vs. non-purple IP (NPIP) tissues, along with linkage and phylogenetic data, allowed an initial identification of 28 candidate genes, 19 of which were further evaluated by RT-qPCR in independent root samples of 3242 and B7262, revealing 15 genes consistently upregulated in the POP in both genetic backgrounds, and two genes upregulated in the POP in specific backgrounds. These include seven transcription factors, seven anthocyanin structural genes, and two genes involved in cellular transport. Altogether, our results point at *DcMYB7*, *DcMYB113*, and a MADS-box (DCAR_010757) as the main candidate genes conditioning *ROPAP* in 3242, whereas *DcMYB7* and MADS-box condition *RIPAP* in this background. In 7262, *DcMYB113* conditions *ROPAP*.

## 1. Introduction

Anthocyanins are flavonoid water-soluble secondary metabolites that serve various roles in plants. They give color to different organs of many vegetables and fruits species and are involved in the attraction of animals and insects for seed dispersal and pollination. These pigments also confer protection against ultraviolet and high-intensity light and can ameliorate abiotic and biotic stresses, including drought, wounding, cold temperatures, and phytopathogen attacks [1,2]. In addition, anthocyanins are beneficial to human health, and the consumption of anthocyanin-rich fruits and vegetables is associated with various health benefits, including a lower incidence of cardiovascular disease, diabetes, arthritis, and some types of cancers, as well as aiding in the prevention of cognitive decline and neurological disorders (reviewed by [3]). Their potent antioxidant and anti-inflammatory properties are believed to play a major role in these health-promoting effects. In addition to their consumption through fresh fruits and vegetables, anthocyanins are used as natural colorants in the food industry, as an alternative to some synthetic food dyes that may pose potential health risks, thereby providing color and increased nutritional value to foods and beverages (reviewed by [4]).

The anthocyanin biosynthetic pathway in plants involves structural genes, encoding the enzymes that participate in the formation of these pigments, and regulatory genes, which control the transcription of the former [5]. In carrot, two simply-inherited loci conditioning root anthocyanin pigmentation, *P*_1_ and *P*_3_, have been described and mapped. *P*_1_ conditions root pigmentation in the genetic background of B7262, a purple-rooted carrot of eastern Turkish origin [6,7,8], whereas *P*_3_ conditions pigmentation in the root and petioles, in the genetic backgrounds of P9547 and PI 652188, two carrot lines from central Turkey and China, respectively [9]. By means of comparative mapping, it was revealed that *P*_1_ and *P*_3_ correspond to different loci located in chromosome 3 and distanced at ~ 30 cM [9]. In recent years, anthocyanin regulatory genes have been identified in these two regions, and some candidate genes were functionally characterized [10]. By means of fine mapping, structural analysis of the carrot genome sequence, and gene expression evaluations in purple vs. non-purple tissues, Iorizzo et al. [11] identified a cluster of six MYB transcription factors in the *P*_3_ region (denominated *DcMYB6* to *DcMYB11*), indicating *DcMYB7* as a key gene controlling anthocyanin pigmentation in the root and petioles, and *DcMYB11* being involved in petiole pigmentation. Later, Bannoud et al. [12], using a similar genetic background as Iorizzo et al. [11], found that *DcMYB7* was not only differentially expressed between purple and non-purple roots, but its expression level was positively correlated with anthocyanin concentration in purple roots with varying pigment intensities, suggesting that this MYB regulates both the presence/absence and the concentration of anthocyanins in the carrot root. Finally, *DcMYB7* and *DcMYB6* were functionally characterized by Xu et al. [13] using a transgenic approach, finding that overexpression of *DcMYB7*, but not *DcMYB6*, induced anthocyanin accumulation in an orange-rooted carrot, whereas *DcMYB7*-knockout lines of the solid purple-rooted cultivar ‘Deep Purple’ exhibited yellow roots. Thus, their study confirmed the role of *DcMYB7* as a key gene controlling root anthocyanin pigmentation. In the *P*_1_ region, another MYB gene, namely *DcMYB113,* was recently described as a key regulatory gene controlling root anthocyanin pigmentation in the carrot cultivar ‘Purple Haze’ [14].

Studies of global transcriptome analysis comparing purple vs. non-purple carrot roots have identified several differentially expressed regulatory genes from the MYB, bHLH, and WD families located throughout the entire carrot genome, and with expression levels correlated with anthocyanin concentration, suggesting that other transcription factors unrelated to the *P*_1_ and *P*_3_ regions may influence anthocyanin pigmentation in the carrot root [15,16]. An anthocyanin vacuolar transporter (*DcGST1*) was also upregulated in solid purple roots compared to orange roots, suggesting a role for this gene in the vacuolar accumulation of these pigments [16].

After their synthesis, anthocyanins may undergo chemical modifications, including glycosylation and acylation, which confer stability to these pigments. In addition to the *P*_1_ and *P*_3_ regions, which harbor MYB genes conditioning anthocyanin biosynthesis, a simply inherited locus conditioning the percentage of root anthocyanin acylation (*Raa1*) was first described and mapped by Cavagnaro et al. [9]. Recently, this genomic region was characterized in detail by Curaba et al. [17] by means of fine mapping, structural analysis of the *Raa1* locus, and gene expression analysis; identifying an acyltransferase gene, *DcSCPL1* (also called *DcSAT1* by [13]), as the main candidate for *Raa1* and the primary regulator of anthocyanin acylation in the carrot root. Additionally, two glycosyltransferase genes (*DcUCGalT1* and *DcUCGXT1*) with expression levels positively correlated with anthocyanin biosynthesis in the carrot root, and unrelated to the *P*_1_ and *P*_3_ regions, have been described by Xu et al. [13,18].

Nearly all of the previous studies concerning anthocyanin pigmentation in the carrot root have compared purple vs. non-purple roots, regardless of the intra-root tissue-specific pigmentation patterns of the carrot lines used, and this has led to the identification of genes conditioning the presence or absence of pigmentation, as well as genes for anthocyanin acylation and glycosylation, in this organ (reviewed by [10]). Although this recently-emerged body of knowledge has undoubtedly advanced our understanding of the genetics underlying carrot anthocyanins, the genetic regulation of these pigments at a tissue-specific and genotype-specific level is still poorly understood. Very few genetic factors conditioning the quantitative accumulation of anthocyanins (i.e., not just their presence/absence) have also been reported to date.

In a recent study, Bannoud et al. [12] investigated the genetic control of tissue-specific anthocyanin pigmentation in the root phloem and xylem, finding that both genomic regions *P*_1_ and *P*_3_ condition pigmentation in the phloem, whereas only *P*_3_ conditions pigmentation in the xylem. Additionally, candidate genes in each of these regions were identified for phloem pigmentation, with *DcMYB7* (in the *P*_3_ region) conditioning both presence/absence and concentration of anthocyanins and two cytochrome CYP450 genes with putative flavone synthase activity (in the *P*_1_ region) negatively influencing pigment concentration. Although this study identified genes affecting pigmentation in the root phloem as a whole (i.e., any degree of purple color observed in this tissue was considered positive for phloem anthocyanins, regardless of the varying anthocyanin-pigmentation patterns observed within this tissue in their mapping population), no distinction or comparison was made between purple and non-purple tissues within the phloem. Thus, the factors conditioning intra-phloem pigmentation patterns, particularly with regards to pigmentation in the outer phloem (also called the cortex) and inner phloem, remain unknown. 

The purple carrot germplasm exhibits ample genetic diversity for anthocyanin pigmentation (reviewed by [19]). In the root, anthocyanin can be present in the epidermis, phloem, and xylem, exhibiting various patterns of pigmentation within each of these tissues, from fully pigmented to not pigmented at all (Supplemental Figure S1). In the phloem, the main tissue influencing total pigment levels in the carrot root, anthocyanin pigmentation varies among genotypes in both pigment concentration and distribution across the phloem tissues, ranging from a few pigmented cells in the outer phloem to a solid-black appearance across the entire phloem. It must be noted that only a very small proportion of the purple carrot germplasm has solid black roots with the presence of anthocyanins throughout the entire phloem and xylem tissues. Thus, from a breeding perspective aiming at increasing total pigment content in carrot roots (e.g., for the production of natural food dyes), it is important to understand the genetic basis of such tissue-specific phenotypic variation. 

In the present study, we investigated the genetic factors conditioning differences in anthocyanin pigmentation patterns within the root phloem of purple carrots. To this end, we performed linkage mapping of purple pigmentation in the outer phloem (OP) and inner phloem (IP), independently, using an F_2_ mapping population segregating for both traits, followed by comparative transcriptome (RNA-Seq) analysis of ‘purple outer phloem’ (POP) vs. ‘non-purple inner phloem’ (NPIP) tissues to identify candidate genes. Finally, transcript levels of 19 candidate genes were analyzed by RT-qPCR in POP and NPIP tissues of two contrasting genetic backgrounds, as well as in OP and IP tissues of solid-purple roots from commercial cultivars. 

## 2. Materials and Methods

### 2.1. Plant Materials

An F_2_ population, denominated ‘3242-F_2_′ (*n* = 254) segregating for presence/absence of purple color in the outer phloem (OP) (also called “cortex”) and inner phloem (IP) tissues of the root, described previously by Bannoud et al. [12], was used for the genetic mapping of anthocyanin pigmentation in OP and IP. This population was obtained from an initial cross between the orange-rooted carrot line ‘Flavor Select’ and a purple-rooted carrot derived from the cross ‘(B2566B × Turkish) × B9304′. The ultimate purple root source in this cross, referred to as ‘Turkish’, exhibits purple OP, IP, and xylem and is a sibling of inbred P9547 (from Central Anatolia, Turkey), the genetic background in which *P*_3_ was first described [9]. B2566 and B9304 are orange-rooted inbreds from diverse European sources.

Carrot roots with different purple pigmentation phenotypes in their OP and IP tissues were selected from two F_2_ sibling populations and an F_3_ derivative population of 3242-F_2_, and used for transcriptomic and RT-qPCR analyses, respectively. Additionally, roots from B7262, a Turkish carrot line with purple OP and non-purple (orange) IP, and genetically unrelated to 3242-F_2_, were also used in RT-qPCR for quantification of transcript levels. 

Finally, two purple carrots with intense anthocyanin pigmentation in both phloem tissues (OP and IP), namely the inbred line P9547 and ‘Pusa asita’, a commercial open-pollinated cultivar from India, were used for validating the expression of candidate genes conditioning pigmentation in these tissues. 

### 2.2. Genetic Mapping of Anthocyanin Pigmentation in the Root Outer and Inner Phloem

Anthocyanin pigmentation in the OP and IP, scored as presence or absence of purple color in these tissues, was mapped as binary traits in the 3242-F_2_ population using the R package R/qtl with the Haley–Knott regression method [20]. These traits were denominated ‘root outer phloem anthocyanin pigmentation’ (*ROPAP*) and ‘root inner phloem anthocyanin pigmentation’ (*RIPAP*). The genetic map was constructed with GBS data from 181 F_2_ individuals, using methods and parameters previously described by Bannoud et al. [12]. Briefly, from a total of 28,473 GBS markers initially detected, after filtering out those with ≥20% of missing data and eliminating redundant markers (i.e., only one marker per recombination event was retained) and markers that deviated significantly from the expected 1:2:1 codominant segregation ratio, 989 non-redundant SNP markers were used to construct nine carrot linkage groups, at LOD > 10, using the software Joinmap 4.0 [21]. Genetic distances among loci were calculated according to Haldane’s function. The carrot genome assembly v2.0 (GenBank accession LNRQ01000000; [22]) was used as a reference to identify marker locations and anchor the linkage groups to the carrot chromosomes.

### 2.3. RNA Sequencing and Transcriptome Analysis

For transcriptomic analyses and identification of differentially expressed genes in purple OP vs. non-purple IP comparisons, three sets of 14-week old carrot roots derived from two sibling families of the 3242-F_2_ population were used. Each sample set was composed of three roots (i.e., three biological replicates) with similar color phenotype, with different sample sets varying in their anthocyanin concentration in the OP tissues. The phenotypes of the three sample sets were labeled: (1) dark purple sample set 1(dP1), with high anthocyanin concentration in the OP (2.45 ± 0.79 mg/g fresh weight) and non-purple IP and xylem; (2) dark purple sample set 2 (dP2), with high anthocyanin concentration in the OP (3.03 ± 0.7 mg/g fw), non-purple IP, and purple xylem; and (3) pale purple (pP), with low anthocyanin content in the OP (0.14 ± 0.1 mg/g fw) and non-purple IP and xylem. Supplementary Figure S2 shows root phenotypes of the three sample sets. Carrot plants of dP1 and pP were grown at the University of California Desert Research and Extension Center, El Centro, California, USA, in 2016, whereas dP2 plants were grown at the University of Wisconsin–Madison Hancock Agricultural Research Station, Wisconsin, USA, in 2016. Samples for RNA-seq were collected from taproot discs from the central portion of the root. Purple OP and non-purple IP tissues were individually dissected from each carrot root and immediately frozen in liquid nitrogen for RNA extraction. Samples names and phenotypes are described in Supplementary Table S1.

Total RNA was extracted from the purple OP and non-purple IP of each carrot root of the dP1, dP2, and pP sets using the TRIzol^®^ Plus RNA Purification Kit (Life Technologies, Carlsbad, CA, USA) following the manufacturer’s protocol. DNA was removed with the ‘DNA free-kit’ provided with the RNA purification kit. RNA quantification was done in a Nanodrop One Spectrophotometer (ThermoFisher Scientific, Waltham, MA, USA), and quality control was done in an Agilent 2100 Bioanalyzer RNA NanoChip. RNA samples were sent to the University of Wisconsin Biotechnology Center for preparation of the libraries and RNA sequencing. For each tissue sample, single-end libraries were prepared and sequenced on an Illumina HiSeq 2000 system using 1 × 100 nt reads. After quality control with FastQC (http://www.bioinformatics.babraham.ac.uk/projects/fastqc/, accessed date: 23 September 2019), reads were filtered with Trimmomatic version 0.32 [23] with adapter trimming and using a sliding window length ≥ 50 and Phred quality score ≥ 28. Reads for each gene available from the V1.0 gene annotation of the carrot genome [22] were mapped and quantified with RSEM [24], using Bowtie2 as an aligner [25]. Pairwise transcriptome comparisons between purple OP and non-purple IP from the same root were performed. Differentially expressed genes (DEGs) were identified in all the pairwise comparisons with the R package edgeR (version 3.20.9) [26] using R version 3.4.1. Filtering parameters were set to only retain transcripts with at least one count per million reads in half of the samples in each pairwise comparison (i.e., in three samples), and the normalization factor was calculated for each sample with the trimmed mean of M-values (TMM) proposed by Robinson and Oshlack [27]. Statistical analyses were done fitting a gene-wise negative binomial generalized linear model (fitGLM) [28]. A false discovery rate (FDR) < 0.05 was set as a threshold for DEGs. Comparisons between samples and normalization factors are presented in Supplementary Table S2.

### 2.4. Search of Candidate Genes Conditioning Anthocyanin Pigmentation in the Root OP and IP 

In order to find candidate genes controlling anthocyanin pigmentation in the root OP and IP, the following criteria were considered: (1) consistent differential expression across all the transcriptome comparisons of ‘purple OP’ vs. ‘non-purple IP’; (2) localization of DEGs in the same map region where the OP and IP pigmentation traits (i.e., *ROPAP* and *RIPAP*, respectively) were mapped; (3) functional relationship of the DEGs with the biosynthetic pathway, modification, and/or transport of anthocyanins; and (4) validation of the DEGs that met the first three criteria, by Reverse Transcription quantitative PCR (RT-qPCR) analysis in additional root samples and genotypes (see below methods for RT-qPCR analysis). The DEGs fulfilling all four criteria were considered major candidates conditioning anthocyanin pigmentation in the root OP and IP tissues. 

Additionally, searches for other candidate genes influencing anthocyanin pigmentation in the root OP and IP and located elsewhere in the genome (i.e., not colocalized in the linkage map with the phenotypic traits *ROPAP* and *RIPAP*) were performed. These included DEGs that fulfilled criteria 1, 3, and 4 (i.e., consistent differential expression in the transcriptome analysis, functionally related to anthocyanin metabolism, and validation by RT-qPCR), as well as genes previously reported to condition anthocyanin pigmentation in other carrot genetic backgrounds. 

### 2.5. Phylogenetic Analysis

Based on results from the transcriptome comparisons, additional analysis was performed on the DEGs belonging to transcription factor (TF) families known to be involved in anthocyanin biosynthesis, regardless of their position in the genome. Thus, carrot sequences from consistently-DEGs belonging to six TF families (MYB, bHLH, Ethylene Response Factor (ERF), NAC, and WRKY) were used in phylogenetic analyses along with gene sequences of these TFs from other plant species to infer the putative role of these DEGs in carrot anthocyanin pigmentation. Phylogenetic trees were constructed with the Neighbor-joining method, Poisson model, and 1000 bootstrap replicates as parameters in MEGA X. Carrot TFs that clustered together with anthocyanin-related genes from other plant species were considered candidate genes. For this, functional subgroups within each of these TF families were considered, as reported previously for bHLH (Pires and Dolan [29], Feller et al. [30]), AP2/ERF (Nakano et al. [31]), and WRKY (Amato et al. [32]).

### 2.6. Real-Time Quantitative Reverse Transcriptase PCR

Transcript levels of 19 candidate genes were quantified by RT-qPCR analysis in the root purple OP and non-purple IP tissues in two unrelated carrot genetic backgrounds with similar color phenotypes in their OP and IP tissues. To this end, an F_3_ derivative population of 3242-F_2_, and the inbred line B7262 with purple OP and non-purple (orange) IP and xylem, were grown at the experimental field of the Horticulture Institute at the National University of Cuyo, Mendoza, Argentina, in 2019. B7262 is the purple-root progenitor of the segregating populations used for the first description [6] and mapping of *P*_1_ [7,8]. The OP and IP tissues were individually dissected from root mid-sections of 12-week old plants, immediately frozen in liquid nitrogen and stored at −80 °C, and later used for RNA isolation and anthocyanin quantitation. Three roots of each genetic background were sampled. Total RNA was extracted from 100 mg fresh weight following the protocol of [33], and the extracted RNA was purified using the SV Total RNA Isolation System Kit (Promega, Madison, WI, USA) according to the manufacturer’s instructions. Total RNA was quantified using an ‘AmpliQuant AQ-07 Nucleic Acid Photometer’ spectrophotometer, and its integrity was checked visually by 1% agarose gel electrophoresis. cDNA was synthesized from 1 µg of RNA by Invitrogen™ SuperScript™ III First-Strand Synthesis System (Thermo Fisher Scientific, MA, USA) and diluted 25-fold for RT-qPCR analysis. RT-qPCR reactions were carried out using the SYBR™ Green PCR Master Mix (Thermofisher Scientific) in a StepOne™ Real-Time PCR system (Applied Biosystems, Foster City, CA, USA) using the following amplification conditions: the first step of enzymatic activation at 95 °C for 10 min, followed by 40 cycles of 95 °C for 15 s (denaturation) and 60 °C for 1 min (annealing and elongation). Analysis of melting curves was performed to verify the amplification specificity. To this end, the dissociation curve protocol started immediately after amplification and consisted of 1 min at 60 °C followed by steps of 80–10 s with temperature increments of 0.3 °C at each step, until reaching 95 °C. Reactions were run in triplicates. Relative quantification of gene expression was performed according to Pfaffl [34] with the modification proposed by Ruijter et al. [35], which takes into account the amplification reaction efficiencies (qPCR efficiencies were calculated from raw data with the software LingReg PCR [36]) using the ‘Differential Gene Expression Analysis’ package from fgStatistics Software [37]. The actin gene was used as an internal control to normalize the variability in expression levels [38]. By this means, we obtained the fold changes in gene expression relative to the internal control gene (actin), and the data were expressed as the ratio ∆Cq-target gene/∆Cq-actin. For most genes, primers for RT-qPCR were designed with Primer3 (v. 4.0.0) software [39] following general considerations for qPCR primer design [40], based on carrot mRNA sequences for each gene aligned with the respective genomic sequences (to avoid intron/exon splice junctions), available in our laboratory. Primers for *DcMYB6* were as previously designed by Iorizzo et al. [11], and primers for *DcbHLH3*, *DcSCPL1*, and *DcUCGXT1* were as reported by Xu et al. [13]. Information on the primers used for RT-qPCR analysis is presented in Supplementary Table S3. Anthocyanin levels in the OP of the 3242 and B7262 root samples used for qPCR analyses were 1.22 ± 0.29 and 1.02 ± 0.09 mg/g fw, respectively, whereas their IP tissues had negligible amounts of pigments (<0.002 mg/g fw).

### 2.7. Anthocyanin Quantification

Carrot root samples used for RNA-seq and qPCR were analyzed for anthocyanin concentration. Anthocyanins were extracted from pigmented OP and IP and non-pigmented IP tissues and quantified by HPLC analysis as described previously [9]. A commercial standard of cyanidin (Sigma-Aldrich, Atlanta, GA, USA) was used for quantitation purposes. Anthocyanin content was expressed as mg of cyanidin equivalents per gram of fresh weight (mg/g fw). 

## 3. Results

### 3.1. Genetic Mapping of Phenotypic Pigment Traits

**Figure 1:**
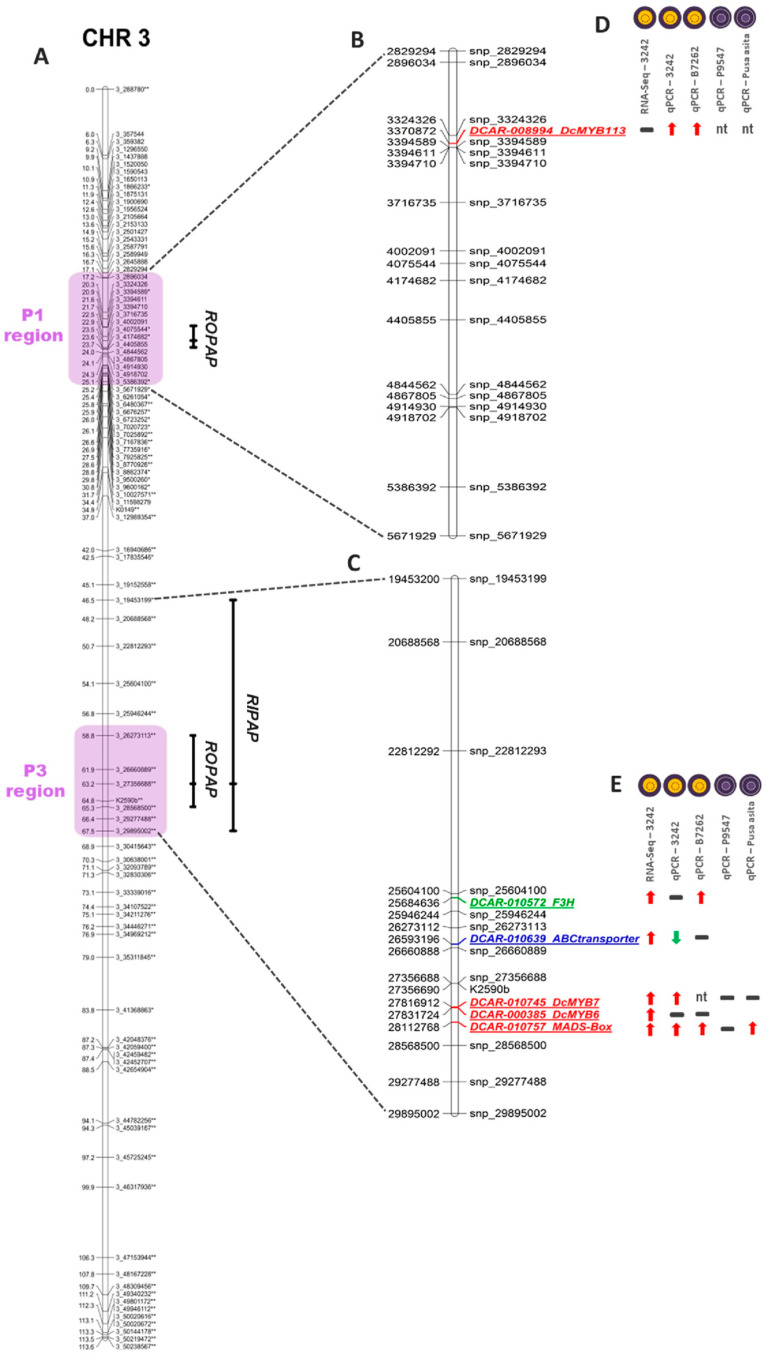
Genetic mapping of the phenotypic traits ‘root outer phloem anthocyanin pigmentation’ (*ROPAP*) and ‘root inner phloem anthocyanin pigmentation’ (*RIPAP*) in the 3242-F_2_ population (**A**). Bars indicate the 1.5 LOD support interval and the position of the maximum LOD value for each binary trait. The *P*_1_ and *P*_3_ regions in chromosome 3, as delimited by Bannoud et al. [12], are highlighted in purple. Asterisks indicate markers that were significantly distorted from the expected segregation ratio for codominant markers in an F_2_ population, at *p* < 0.05 (*) and *p* < 0.01 (**). Magnified images correspond to the physical map (in nucleotide distances) of the *P*_1_ (**B**) and *P*_3_ (**C**) regions associated with *ROPAP* and *RIPAP*, and include candidate regulatory (in red letters) and structural genes involved in anthocyanin biosynthesis (green) and transport (blue), along with their performance in different gene-expression experiments and genetic backgrounds (**D**,**E**). Red and green arrows indicate up and downregulation in the outer phloem relative to the inner phloem, respectively, whereas black dashes indicate not differentially expressed. nt: No transcripts detected. Root section phenotypes of the different genetic backgrounds analyzed are illustrated for each gene expression experiment.

Anthocyanin pigmentation in the OP and IP, phenotyped as presence or absence of purple color in each tissue (binary traits), was genetically mapped in the 3242-F_2_ population. These phenotypic traits were called *ROPAP* (for ‘root outer phloem anthocyanin pigmentation’) and *RIPAP* (for ‘root inner phloem anthocyanin pigmentation’), respectively. Loci for both traits colocalized with the previously described *P*_1_ and *P*_3_ regions in chromosome 3, conditioning anthocyanin pigmentation in different carrot genetic backgrounds (Figure 1 and Table 1). Two significant loci for *ROPAP* co-localized with the *P*_1_ and *P*_3_ regions, whereas a single locus conditioning *RIPAP* co-localized with *P*_3_. These results suggest that in 3242-F_2_ both regions, *P*_1_ and *P*_3_, control anthocyanin pigmentation in the outer phloem, whereas pigmentation in the inner phloem is conditioned by both a single and broader genomic region that includes *P*_3_.

### 3.2. Transcriptome Comparisons

#### 3.2.1. Differentially Expressed Genes in the ROPAP and RIPAP Regions

Given that *ROPAP* and *RIPAP* colocalized with *P*_1_ and *P*_3_ in Chr 3, and the maximum LOD value of the formers coincided with the center of the *P*_1_ and *P*_3_ regions, as defined by Bannoud et al. [12], we searched for candidate genes conditioning *ROPAP* in the region of *P*_1_ (delimited by genome coordinates 2.89–5.38 Mb) and *P*_3_ (26.3–29.9 Mb), whereas candidates for *RIPAP* were searched for in the genomic region (19.45–29.89 Mb) overlapping the 1.5 LOD support interval of the trait, which includes *P*_3_ (Figure 1). The resulting data from the analysis of DEGs in these three regions are presented in Supplementary Tables S4–S6 and Supplementary Figure S3.

In the *P*_1_ region, 37 DEGs were identified in POP vs. NPIP transcriptome comparisons in the dark purple ‘dP1′ subset of samples, 23 DEGs in the dark purple ‘dP2′ samples, and 19 DEGs in the pale purple ’pP’ samples (Supplementary Tables S4 and S5). Three of these DEGs were consistently upregulated across all the pairwise comparisons and included two glycerophosphodiester phosphodiesterase-like genes (DCAR_009117 and DCAR_009118) and a chloroplastic NADH pyrophosphatase (DCAR_009201) (Supplementary Table S6). None of these genes are directly related to anthocyanin metabolism. 

In the *P*_3_ region, 39 DEGs from POP vs. NPIP comparisons were identified in dP1, 38 DEGs in dP2, and 20 DEGs in pP root samples. Eleven of these DEGs were consistently upregulated (8 DEGs) or downregulated (3 DEGs) in POP (relative to NPIP) across all the pairwise comparisons (Supplementary Table S6). The consistently upregulated genes include a ‘vacuolar transporter belonging to the ATP-Binding Cassette family (ABC proteins) class C’ (DCAR_010639); a ‘plastidial pyruvate kinase’ (DCAR_010645); a ‘cationic peroxidase 1′ (DCAR_010688); a ‘pathogen-related protein’ (DCAR_010700); two carrot MYB transcription factors, *DcMYB7* (DCAR_010745) and *DcMYB6* (DCAR_000385); a ‘MADS-box transcription factor’ (DCAR_010757); an ‘Irregular Xylem (IRX)-like protein’ associated with xylan deposition in secondary cell walls (DCAR_010869). The three DEGs consistently downregulated in POP were: a ‘ribose-phosphate pyrophosphokinase’ (DCAR_010690); a member of the ‘multidrug and toxic compound extrusion (MATE)’ family of cellular and organelle transporters (DCAR_010718); and a ‘probable xyloglucan endotransglucosylase/hydrolase protein’ (DCAR_010780). Of these genes, *DcMYB7* and *DcMYB6* have been previously implicated with anthocyanin regulation in carrot roots [11,12,13,41]; and anthocyanin-specific MADS-box transcription factors have been proved to regulate the biosynthesis of these pigments in some fruits [42]. Members of the MATE and ABC-C transporter families are also known to be involved in the transport and uptake of anthocyanins into the vacuole in other plant species [43,44]. However, the carrot MATE gene identified in the *P*_3_ region was unexpectedly consistently downregulated in the POP, suggesting that it is not involved in the pigmentation of this tissue and was therefore excluded as a potential candidate. The putative (annotated) functions of the rest of the DEGs consistently identified in the *P*_3_ region are, apparently, unrelated to anthocyanin metabolism.

In the *RIPAP* region conditioning pigmentation in the inner phloem, 65 DEGs were identified in dP1 carrot roots, 62 DEGs in dP2, and 38 DEGs in pP roots. Nineteen of these DEGs were consistently upregulated (14 DEGs) or downregulated (5 DEGs) in the POP tissues (relative to NPIP) across all the pairwise comparisons (Supplementary Table S6). Because the support interval of *RIPAP* includes *P*_3_, most of the DEGs identified correspond to the latter region and were described above. However, eight additional DEGs were consistently identified in the *RIPA*P region outside *P*_3_ (between coordinates 19.4 and 26.3 Mb). Six of these DEGs were upregulated in the POP, and included a ‘flavanone 3-hydroxylase’ (DCAR_010572); a member of the ‘aldehyde dehydrogenase family 2′ (DCAR_010446); a ‘mechanosensitive ion channel protein 10′ (DCAR_010498); an ‘inositol-tetrakisphosphate 1-kinase’ (DCAR_010529); an ‘UNC93-like protein’ (DCAR_010563); and a ‘CDP1K-related kinase 4′ (DCAR_010578); whereas the remaining two DEGs were downregulated in POP, and included a ‘SEPALLATA 3′ protein involved in floral development (DCAR_010471), and a ‘YLS9′-like gene associated with disease response and leaf senescence (DCAR_010573). The consistently upregulated F3H was the only gene related to anthocyanin metabolism in this region and was, therefore, considered a potential candidate for *RIPAP*.

#### 3.2.2. Genome-Wide Search of DEGs Associated with Anthocyanin Pigmentation in the Root Outer and Inner Phloem

Besides searching for major candidate genes controlling *ROPAP* and *RIPAP*, which are expected to be located within the confidence interval of the mapped phenotypic traits, a genome-wide search -outside the *ROPAP* and *RIPAP* regions- of DEGs in all the pairwise POP vs. NPIP comparisons was performed to identify additional candidates influencing anthocyanin pigmentation in these root tissues. The number of DEGs found per comparison ranged from 2007 (in pP roots) to 3593 (in dP1 roots), with a total of 5672 non-redundant DEGs identified (Supplementary Table S4). A total of 817 DEGs were consistently revealed across all the comparisons, with 659 DEGs being consistently upregulated and 158 DEGs being downregulated in POP. Further analysis of regulatory and structural anthocyanin genes within this set of consistently-DEGs was performed.

##### Regulatory Genes

A total of 71 DEGs annotated as transcription factors (TF) in the carrot genome were consistently upregulated (56 TFs) or downregulated (15 TFs) in the root POP tissues (Supplementary Table S7). Comparative sequence analysis of these DEGs with anthocyanin-related TF families from other species revealed that 45 of the carrot DEGs belong to the MYB (18 DEGs), WRKY (12), bHLH (7), ERF (6), and NAC-like (2) TF families (Supplementary Table S8). These gene families of TFs participate in multiple and diverse processes in the plant, and some of their members are known to be involved in the regulation of anthocyanin biosynthesis. Thus, in order to infer the possible function of the carrot TFs, we performed phylogenetic analysis for each TF family, using these carrot TFs along with members from other species with known functions (Supplementary Table S9). The results are presented in Supplementary Figures S4–S8.

Phylogenetic analysis of the 18 carrot MYBs and 53 MYBs from other species revealed the clustering of two carrot genes, DCAR_003717 and DCAR_028312, in a clade of MYBs involved in the transcriptional activation of proanthocyanidins and flavonols, whereas another carrot gene (DCAR_001079) grouped in a clade of MYBs acting as anthocyanin repressors (Supplementary Figure S4). Since DCAR_001079 was upregulated in POP relative to NPIP, it is unlikely that this MYB is acting as a repressor in the anthocyanin biosynthetic pathway. The rest of the carrot MYBs did not cluster with other MYBs related to the flavonoid pathway, suggesting that they are not involved in the regulation of anthocyanins, and therefore they were excluded as potential candidate genes. 

Among the four carrot bHLH TFs differentially expressed across all the pairwise POP vs. NPIP comparisons, DCAR_002739 grouped in the same phylogenetic clade as bHLH genes from other species known to be involved in the regulation of anthocyanin biosynthesis (Supplementary Figure S5). This gene, called *DcbHLH3*, is a homolog of the anthocyanin-related apple (*Malus domestica*) *bHLH3,* and was recently reported to be activated by *DcMYB7* in purple carrot roots [13]. The rest of the carrot bHLH genes were not phylogenetically related to other bHLHs associated with anthocyanins. 

Analysis of the six carrots ERF TFs revealed that DCAR_016339 and DCAR_007860 clustered in the same phylogenetic clade as the anthocyanin enhancer *PyERF3*, an ethylene-responsive factor from pear known to co-regulate fruit anthocyanin biosynthesis along with MYB and bHLH TFs from that species [45] (Supplementary Figure S6). Of these two carrot ERFs, only DCAR_016339, annotated as *DcERF1*, had good bootstrap support. Conversely, the inclusion of DCAR_007860 in this clade was not statistically supported (according to its bootstrap value below the threshold of 50), and therefore this gene was discarded as a potential candidate. The rest of the carrot ERFs were phylogenetically unrelated to anthocyanin TFs.

None of the carrot WRKY and NAC DEGs identified in the POP vs. NPIP transcriptome comparisons were phylogenetically related to TFs from other species involved in anthocyanin metabolism (Supplementary Figures S7 and S8), suggesting that they are not involved in carrot anthocyanin pigmentation. Thus, they were discarded as candidate genes.

##### Structural Anthocyanin Biosynthetic Genes

Among the genes annotated in the carrot genome as flavonoid or anthocyanin structural biosynthetic genes by Iorizzo et al. [22], 13 DEGs were consistently upregulated in POP in all of the pairwise POP vs. NPIP transcriptome comparisons (Supplementary Table S10). These genes encode the following enzymes of the anthocyanin pathway: three phenylalanine ammonia-lyases (PAL) (DCAR_014989, DCAR_017697, DCAR_020833), two UDP-glycosyltransferases (UDPGT) (DCAR_009912, DCAR_019074), two 4-coumaric acid:coenzyme A ligases (4CL) (DCAR_025617, DCAR_021385), a leucoanthocyanidin dioxygenase (LDOX) (DCAR_06772), a flavanone 3-hydroxylase (F3H) (DCAR_009483), a flavanone 3′-hydroxylase (F3′H) (DCAR_014032), a dihydroflavonol reductase (DFR) (DCAR_021485), a chalcone isomerase (CHI) (DCAR_027694), and a chalcone synthase (CHS) (DCAR_030786). 

Three additional structural genes involved in the chemical modification of carrot anthocyanins, namely their glycosylation and acylation, were found to be consistently upregulated in POP tissues across all the transcriptome comparisons. These genes, which were not initially annotated as structural anthocyanin genes in the carrot genome, were recently described. *DcUGalT1* (DCAR_009912) is involved in the first step of cyanidin glycosylation and encodes a carrot galactosyltransferase that catalyzes the transfer of the galactosyl moiety from UDP-galactose to cyanidin [18]. *DcUCGXT1* (DCAR_021269) encodes an ‘anthocyanidin 3-O-glucoside 2′-O-glucosyltransferase’ orthologous to glycosyltransferases from *Arabidopsis* and kiwifruit that catalyze the glycosylation of Cy3G to produce Cy3XG, and was found to be upregulated in purple carrot roots relative to orange roots [13]. *DcSCPL1* (LOC_108214129) is an SCPL-acyltransferase recently described as the primary regulator of anthocyanin acylation in carrot roots [17].

##### Genes Involved in Intracellular Transport of Anthocyanins

Two carrot genes related to anthocyanin transport were consistently upregulated in all the POP vs. NPIP transcriptome comparisons; a glutathione-S-transferase (*DcGST1*) (DCAR_03401) located in Chr 1, and a MATE transporter (DCAR_031151) in Chr 9. Members of the MATE [44] and GST (reviewed by [46]) families are involved in the transport and uptake of anthocyanins into the vacuole in several plant species. In carrot, *DcGST1* was upregulated in purple roots compared to orange roots, suggesting a role for this gene in the vacuolar accumulation of these pigments [16].

### 3.3. Preliminary Identification of Candidate Genes Based on Linkage, Transcriptome, and Phylogenetic Analyses

Based on results from linkage mapping and transcriptome analysis, four major candidates fulfilling the first three criteria for candidate genes (i.e., linkage map colocalization with the phenotypic traits, consistent differential expression in all the POP vs. NPIP transcriptome comparisons, and annotated function related to anthocyanins metabolism) were preliminarily identified in the *P*_3_ region associated with *ROPAP* and *RIPAP*. These were the previously described MYBs *DcMYB7* and *DcMYB6* [11,13,41], a MADS-box TF, and an ABC-C transporter. Additionally, a candidate F3H gene associated exclusively with *RIPAP* was identified. In the *P*_1_ region associated with *ROPAP*, no candidate genes were found. 

Outside the *ROPAP* and *RIPAP* regions, additional candidate genes influencing anthocyanin pigmentation in the outer root phloem and inner phloem tissues were identified. These were genes located elsewhere in the carrot genome that fulfilled criteria one and three of candidate genes (i.e., consistent differential expression in POP vs. NPIP comparisons, and annotated function related to anthocyanins), and included four transcription factors (two MYBs, a bHLH (*DcbHLH3*), and an ERF gene); 13 anthocyanin structural biosynthetic genes (three PAL, two UDPGT, two 4CL, and one each of LDOX, F3H, F3′H, CHI, and CHS); three recently described structural genes involved in the glycosylation (*DcUGalT1* and *DcUCGXT1*) and acylation (*DcSCPL1*) of carrot anthocyanins; and two genes related to anthocyanin transport belonging to the GST (*DcGST1*) and MATE transporter families. 

The identified candidate genes listed above were selected for further analysis by RT-qPCR. In addition, two other genes previously reported to influence anthocyanin pigmentation in carrots or other species were also considered for RT-qPCR evaluations, regardless of their expression patterns in the transcriptome analysis. One of these genes is DCAR_008894, an MYB TF called *DcMYB12* by Bannoud et al. [12] and *DcMYB113* by Xu et al. [13] that was recently demonstrated to be the gene conditioning *P*_1_ in the purple carrot cultivar ‘Purple Haze’ [14]. This gene was not differentially expressed in the POP vs. NPIP transcriptome comparisons in the 3242-F_3_ background. The second gene is sucrose invertase (DCAR_009482), located near the boundary of the *P*_1_ region, which was previously found to be upregulated in purple carrot roots relative to non-purple roots [12]. In the present study, this gene was upregulated in the POP of dP2 but not in dP1 and pP samples sets. Sucrose invertases, which cleave sucrose into fructose and glucose, have been shown to influence anthocyanin biosynthesis in other plant species [47].

### 3.4. Expression Analysis by RT-qPCR of Selected Candidate Genes in 3242 and B7262 Genetic Backgrounds

**Figure 2:**
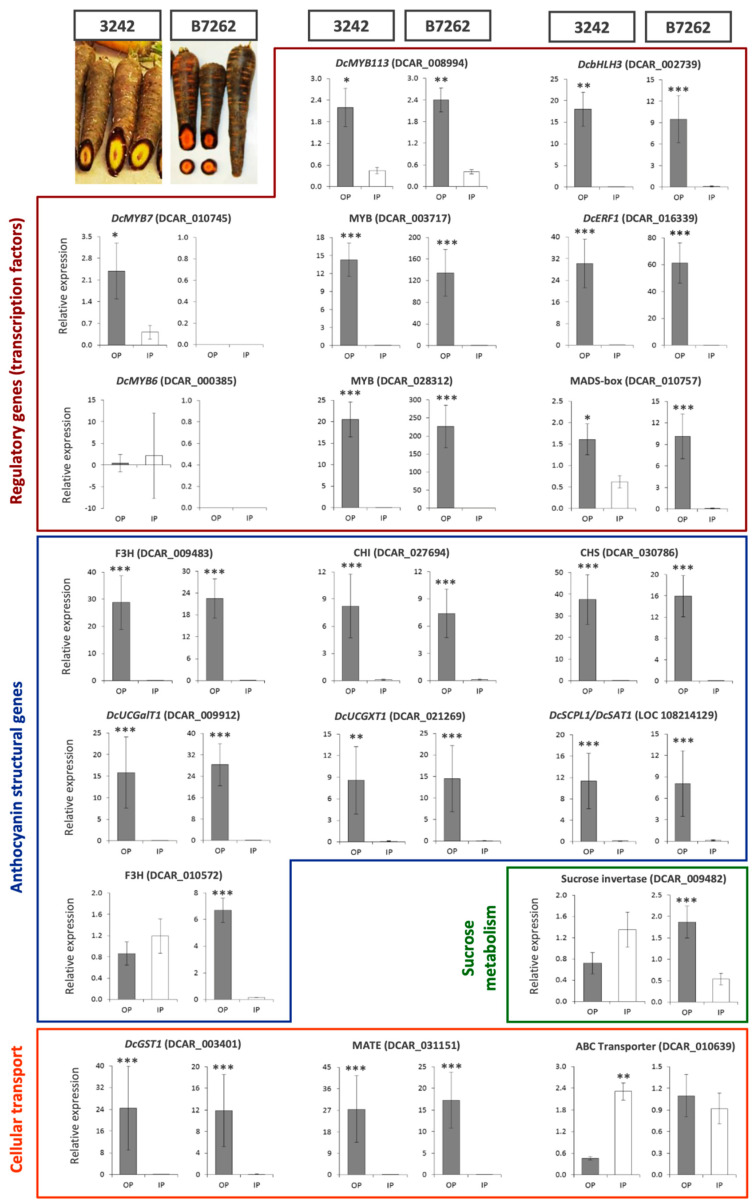
Comparative analysis of transcript levels by RT-qPCR for 19 selected genes in purple outer phloem (OP) (gray bars) vs. non-purple inner phloem (IP) (white bars) tissues from roots of 3242 and B7262 genetic backgrounds. For 3242, roots from a derivative F_3_ family of 3242-F_2_ (used for mapping and transcriptomic analyses) were used. Root phenotypes for both genetic backgrounds are shown in the upper-left images. The selected anthocyanin-related genes were consistently differentially expressed in all of the POP vs. NPIP transcriptome comparisons (16 genes), and/or they colocalized with *ROPAP* and *RIPAP* in the linkage map, and/or they were previously reported to be involved in the biosynthesis, modification or transport of anthocyanins. Bars indicate mean values for the fold change in gene expression relative to the expression of the internal control gene (actin), as calculated from the ratio ∆Cq-target gene/∆Cq-actin [34] and their standard errors. Asterisks indicate significantly higher gene expression at *p* < 0.05 (*), *p* < 0.01 (**), and *p* < 0.001 (***).

Gene expression analysis by RT-qPCR of 19 preliminarily-selected candidate genes in POP vs. NPIP tissues of roots of 3242-F_3_ (cultivated in a different environment (Mendoza, Argentina) from the samples used in the RNA-Seq analysis) and the inbred line B7262, revealed 14 DEGs consistently upregulated in the POP in both genetic backgrounds (Figure 2). These DEGs include six transcriptional activators belonging to the MYB (3 DEGs), bHLH (1 DEG), MADS-box (1 DEG), and ERF (1 DEG) TF families; six anthocyanin biosynthetic structural genes (F3H, CHS, CHI, two glycosyltransferases (*DcUCGalT1* and *DcUCGXT1*), and one acyltransferase (*DcSCPL1*)); and two genes involved in the intracellular transport and vacuolar uptake of anthocyanins (*DcGST1* and a MATE transporter). Additionally, three genetic background-specific DEGs upregulated in the POP were identified; *DcMYB7* in 3242; and an F3H (DCAR_010572), and a sucrose invertase (DCAR_009482) in B7262. Of the remaining two genes, *DcMYB6* was not differentially expressed in both genetic backgrounds, and the ABC-C transporter gene (DCAR_010639) was unexpectedly overexpressed in the non-purple inner phloem tissues of 3242, while it was not differentially expressed in B7262; therefore, based on these results, these genes were excluded as potential candidates conditioning the differential POP/NPIP pigmentation. 

Major candidate genes conditioning anthocyanin pigmentation in the outer root phloem and inner phloem are expected to be located in the *ROPAP* and *RIPAP* regions of Chr 3, respectively. *DcMYB113,* positionally associated with *ROPAP* in the *P*_1_ region, was consistently upregulated in the POP in both genetic backgrounds (Figure 2). Among the four genes in the *P*_3_ region associated with R*OPAP* and *RIPAP*, *DcMYB7* was upregulated in the POP in 3242 but not in B7262. In the latter, no transcripts were detected for this gene in either POP or NPIP tissues. The MADS-box TF was consistently upregulated in the POP in both genetic backgrounds. The other two genes, *DcMYB6* and the ABC-C Transporter, were not differentially expressed, or their differential expression was not associated with anthocyanin pigmentation, as described above. The F3H gene located within the *RIPAP* confidence interval but outside the *P*_3_ region was upregulated in the POP in B7262 but not in 3242. Altogether, these results exclude *DcMYB6* and the ABC-C transporter as potential candidates, leaving three transcription factors (DcMYB113, DcMYB7, and MADS-box) and one structural gene (F3H) as major candidates for ROPAP and RIPAP.

### 3.5. RT-qPCR Analysis of Major Candidate Genes for ROPAP and RIPAP in Fully-Pigmented Carrot Roots 

**Figure 3:**
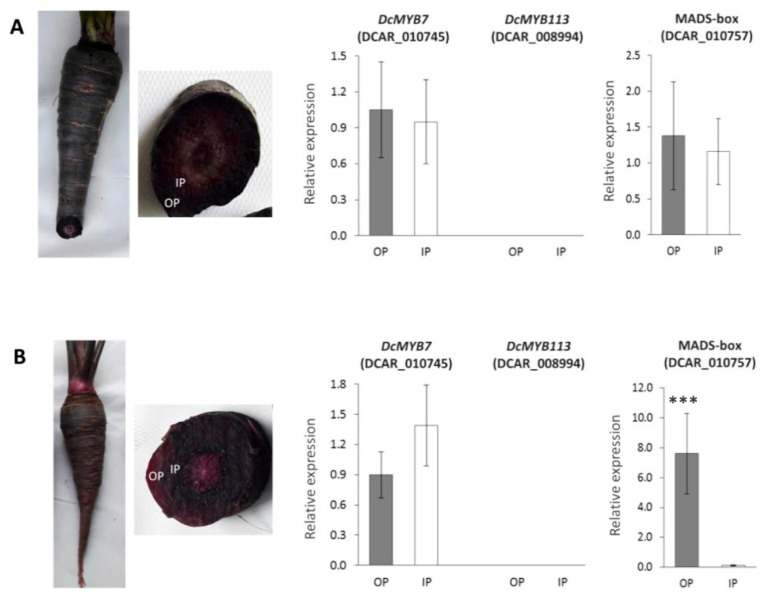
Gene expression analysis (RT-qPCR) of *DcMYB7*, *DcMYB113* and MADS-box (DCAR_010757) in the root purple outer phloem (OP) (gray bars) and purple inner phloem (IP) (white bars) of the carrot inbred line P9547 (**A**), which is genetically related to 3242, and the Indian cultivar Pusa asita (**B**). Root phenotypes with cross-sections indicating the OP and IP tissues, and their gene expression profiles are presented. Bars indicate mean values for the fold change in gene expression relative to the expression of the internal control gene (actin), as calculated from the ratio ∆Cq-target gene/∆Cq-actin [34], and their standard errors. Asterisks (***) indicate significantly higher gene expression at *p* < 0.001, whereas OP vs. IP comparisons without asterisks is not statistically different at *p* ≤ 0.05. The absence of bars for *DcMYB113* indicates that no transcripts were detected.

In order to further confirm the candidacy of the three transcription factors associated with *RIPAP* and *ROPAP*, their gene expression profiles were analyzed in the root purple inner phloem (PIP) and purple outer phloem (POP) in two carrot lines with their phloem tissues fully pigmented; the inbred P9547, genetically related to 3242, and the Indian cultivar ‘Pusa asita’ (Figure 3). In P9547, *DcMYB7* and MADS-box were expressed at similar rates in the PIP and POP, with no significant differences found in transcript levels between both phloem tissues, whereas no transcripts of *DcMYB113* were detected in either POP or PIP (Figure 3A). In Pusa asita, a similar expression pattern (as observed in P9547) was found for *DcMYB7* and *DcMYB113*, whereas the MADS-box gene was upregulated in the POP (*p* < 0.001), despite the fact that both phloem tissues had the same color phenotype (Figure 3B). These data, together with results from previous analyses (described above), suggest that: (1) in P9547, *DcMYB7* and MADS-box condition both *ROPAP* and *RIPAP*; (2) in Pusa asita, *ROPAP* is controlled by *DcMYB7* and MADS-box, whereas only *DcMYB7* conditions *RIPAP*; (3) anthocyanin pigmentation in both of these genetic backgrounds is controlled by the *P*_3_ region, associated with *ROPAP* and *RIPAP*, but not *P*_1_. 

## 4. Discussion

This study investigated, for the first time, the genetics underlying carrot anthocyanin pigmentation at a tissue-specific level within the root phloem. Despite their similar names, the outer phloem (also called the cortex) and the inner phloem are anatomically and ontogenically different from the inner phloem, including remnants of the primary phloem and parenchymal tissue in the outer phloem expanding somewhat later in development [48]. The presence or absence of anthocyanins in the OP and IP are genetically conditioned traits, and the purple carrot germplasm exhibits broad genetic and phenotypic variation for these traits (Supplementary Figure S1). While pigmentation in the OP can occur independently from pigmentation in the IP, the presence of purple IP always occurs in the presence of purple OP. Our mapping results in the 3242-F_2_ population show overlapping map positions of *ROPAP* and *RIPAP* with the previously described *P*_1_ and *P*_3_ genomic regions harboring anthocyanin QTL and simply-inherited loci [8,9,11,12], and indicate that two regions on Chr 3-colocalized with *P*_1_ and *P*_3_- control pigmentation in the outer phloem, whereas another broader region, that includes *P*_3_, conditions pigmentation in the inner phloem (Figure 1). Addressing the tissue specificity of *P*_1_ and *P*_3_ was facilitated by the unusual fact that *P*_1_ and *P*_3_, as well as pigmentation in the outer phloem and inner phloem, all segregated in the same mapping population.

Given the map position of *ROPAP* and *RIPAP*, we used a combination of transcriptome (RNA-seq), phylogenetic, and gene expression (RT-qPCR) analyses to identify major and secondary candidate genes influencing *ROPAP* and *RIPAP* in two carrot genetic backgrounds, namely the 3242-F_2_ mapping population and the inbred line B7262. Major candidate genes were located within the *ROPAP* and *RIPAP* confidence intervals and had predicted functions and consistent expression profiles associated with anthocyanin pigmentation across all the analyses performed, in at least one of the genetic backgrounds; whereas secondary candidates fulfilled similar criteria, but they were located outside the *ROPAP* and *RIPAP* regions (the criteria for candidate genes were described in materials and methods section). On the basis of these criteria, major candidates presume hierarchical genetic control over the expression of these traits, whereas secondary candidates may influence the traits to different extents, most likely interacting with the formers.

Overall, a total of 28 candidate genes associated with *ROPAP* and *RIPAP* were initially identified across all the analyses and plant materials used. These include eight transcription factors; 16 anthocyanin biosynthetic structural genes, including genes involved in anthocyanin glycosylation and acylation; three genes involved in the cellular transport and uptake of these pigments into the vacuole, and one gene related to sucrose metabolism. Supplementary Table S11 presents summarized data for the performance of these genes across the different analyses performed.

Only four of the 28 initially considered genes fulfilled all the criteria for major candidate genes controlling *ROPAP* and *RIPAP*. Thus, these genes (1) were consistently upregulated in the POP (relative to NPIP) in all of the transcriptome comparisons; (2) had genomic positions within the confidence interval of the mapped traits *ROPAP* and *RIPAP*; (3) had predicted functions related to anthocyanin biosynthesis (based on their sequence homology to previously functionally-characterized genes); and (4) validated their overexpression in the POP in additional root samples from 3242 and/or B7262 genetic backgrounds by means of RT-qPCR analysis. Based on these criteria, our results point to *DcMYB7*, *DcMYB113*, and a MADS-box (DCAR_010757) as the main candidate genes conditioning *ROPAP* in 3242, whereas *DcMYB7* and MADS-box condition *RIPAP* in this background. In B7262 roots, which exhibit purple pigmentation only in the outer phloem, D*cMYB113* conditions *ROPAP*.

These data are consistent with previous findings on carrot anthocyanin genetics in backgrounds related to the ones used in this study. In B7262, purple root pigmentation is controlled by *P*_1_, as revealed from inheritance [6] and linkage mapping [7,8] analyses. Very recently, *DcMYB113* was reported as the main candidate gene conditioning anthocyanin pigmentation in the cultivar ‘Purple Haze’, which is phenotypically similar to B7262, suggesting that *DcMYB113* controls *P*_1_ [14]. In the present study, *DcMYB113* was the only gene located within the *P*_1_ region and the confidence interval of *ROPAP* (Figure 1) that was upregulated in the POP of B7262, concomitantly with at least 10 structural genes involved in anthocyanin biosynthesis, chemical modification, and transport (Figure 2), suggesting that *DcMYB113* controls pigmentation in the outer phloem of B7262 by activating the transcription of anthocyanin structural genes, and further confirming its candidacy as the main regulator of *P*_1_. 

As described above, in the 3242 genetic background, both regions, *P*_1_ and *P*_3_, are conditioning anthocyanin pigmentation. At a tissue-specific level, pigmentation in the outer phloem (*ROPAP*) was associated with both of these regions (Figure 1), with three major candidate genes identified: *DcMYB113*, associated with *P*_1_, and *DcMYB7* and MADS-box (DCAR_010757), associated with *P*_3_. 

Pigmentation in the inner phloem (*RIPAP*) was associated with a broader genomic region that includes *P*_3_ (Figure 1), and the two previous genes are major candidates for this trait as well. The fact that both of these genes were consistently downregulated in the non-purple inner phloem when compared to the purple outer phloem of the same roots (Supplementary Table S11 and Figure 2), but no differential expression was found between the purple outer phloem and purple inner phloem of P9547 (Figure 3), which is genetically related to 3242, strengthens the candidacy of *DcMYB7* and MADS-box (DCAR_010757) for the genetic control of *RIPAP* in the 3242 background. However, in the other carrot line with fully-pigmented roots, namely Pusa asita, only *DcMYB7* exhibited the expected not-statistically different expression profile in POP vs. PIP comparisons, with the MADS-box gene being still upregulated in the outer phloem despite the fact that both phloem tissues had intense purple color (Figure 3), indicating that only *DcMYB7* controls pigmentation in the inner phloem in this genotype. Altogether, these results suggest that *DcMYB7* is a key gene conditioning *RIPAP*, whereas MADS-box (DCAR_010757) has a genotype-dependent activity and may regulate or co-regulate *RIPAP* in specific genetic backgrounds (e.g., 3242), but not in others.

The other candidate gene identified within the *RIPAP* confidence interval, F3H (DCAR_010572), was consistently upregulated in the POP in all the ‘POP vs. NPIP’ transcriptome comparisons, but its expression profile was only validated, by qPCR, in the B7262 background, but not in 3242 (Figure 2), suggesting a genotype dependent-activity for this gene. Expression analysis of this gene in the IP of other purple carrot backgrounds revealed inconsistent association with the inner-phloem purple phenotype (data not shown). Thus, although this F3H may be involved in pigmentation of the inner phloem in some genetic backgrounds, it is unlikely that this structural gene is a hierarchical gene conditioning *RIPAP*, but rather may be regulated by *DcMYB7* and/or MADS-box in specific backgrounds.

With a few exceptions in which genotype-specific gene-expression profiles were detected (namely, F3H (DCAR_010572), a sucrose invertase (DCAR_009482), and an ABC transporter (DCAR_010639)), we generally found expression patterns of 17 structural genes that varied concomitantly with the expression of *DcMYB7*, *DcMYB113* and MADS-box (DCAR_010757) (Figure 2 and Supplementary Table S11), suggesting that these major candidate genes act as transcriptional activators upon the same structural genes to regulate *ROPAP* and *RIPAP* in their respective genetic backgrounds (3242 and B7262). These include anthocyanin biosynthetic genes, glycosyl- and acyltransferases that mediate pigment glycosylation and acylation for increased chemical stability, and genes involved in their cellular transport and accumulation into the vacuole. In addition to the structural genes, four transcription factors (*DcbHLH3*, MYB (DCAR_003717), MYB (DCAR_028312), and *DcERF1*) were concomitantly expressed with key candidate genes (*DcMYB7*, *DcMYB113*, and MADS-box) in both genetic backgrounds, suggesting that they are transcriptionally regulated by the latter. The possibility that they may act independently from the latter, regulating anthocyanin pigmentation in the outer and inner phloem tissues, is unlikely given that only *DcMYB7*, *DcMYB113*, and MADS-box had colocalized map positions with *ROPAP* and *RIPAP.* Additional support for the hierarchical role of *DcMYB7* and *DcMYB113* comes from previous studies demonstrating that both of these genes were able to activate the expression of *DcbHLH3*, as well as the glycosyltransferase *DcUCGXT1* and the acyltransferase *DcSAT1* [13,14]. Co-expression between *DcMYB7* and several of the structural genes considered as secondary candidates of *ROPAP* and *RIPAP* in the present work was also found in earlier studies [11,12,16,17].

The role of MADS-box (DCAR_010757), positionally associated with *ROPAP* and *RIPAP* in the *P*_3_ region is still inconclusive. Based on its position and expression profiles in the transcriptome and qPCR analyses, this gene appears as a major candidate. In addition, in a previous study, this gene colocalized with root anthocyanin QTL, and its expression was upregulated in purple roots relative to non-purple (orange) roots [12].

MADS-box transcription factors have also been proved to regulate anthocyanin biosynthesis in several species, including sweet potato [49], pear [50], bilberry [42], and *Arabidopsis thaliana* [51]. However, in the present study, this MADS-box was not phylogenetically related to MADS-box genes from other species known to be involved in anthocyanin biosynthesis (Supplementary Figure S9), and its expression in the POP and PIP in roots of Pusa asita was not associated with the color phenotype (Figure 3). Thus, further analysis is required [e.g., by testing its regulatory activity upon structural anthocyanin genes, in a similar way as done for *DcMYB7* and *DcMYB113* [13,14]] to conclusively elucidate the role of this gene in carrot anthocyanin pigmentation. 

## Figures and Tables

**Table 1:** Genetic mapping of binary phenotypic traits ‘root outer phloem anthocyanin pigmentation’ (*ROPAP*) and ‘root inner phloem anthocyanin pigmentation’ (*RIPAP*).

Rait	Chr.	Max LOD Position ^†^	Nearest Marker	Position of Nearest Marker (cM)	Max. LOD	1.5 LOD Interval (nt)	Difference LOD Interval (nt)	1.5 LOD Interval (cM)	Difference LOD Interval (cM)	% Variance Explained
*ROPAP*	3	3_4002091	3_4002091	22.9	17.4	3,394,611–4,075,544	680,933	21.6–23.5	1.9	23.6
*ROPAP*	3	Chr3.loc63	3_27356688	63.2	13.9	26,273,113–28,568,500	2,295,387	58.8–65.3	6.6	17.8
*RIPAP*	3	3_27356688	3_27356688	63.2	5.3	19,453,199–29,895,002	10,441,803	46.5–67.5	21.1	14.7

^†^ Refers to the map position of the maximum LOD value for each trait. 1.5 LOD intervals are expressed in physical (nt) and genetic distances (cM).

## Data Availability

Data available in a publicly accessible repository that does not issue DOIs. Publicly available datasets were analyzed in this study. These data can be found here: NCBI GEO number GSE181611.

## Supplementary Materials

The following are available online at https://www.mdpi.com/article/10.3390/genes12101464/s1, Supplementary Figures S1–S9 and Supplementary Tables S1–S11. Supplementary Figure S1. A. Cross-section of a carrot root from the 3242-F2 mapping population, indicating the tissues that segregate for anthocyanin pigmentation in this background. B-G. Examples of the range of genetic variation in the carrot germplasm for anthocyanin pigmentation in different root tissues. Images B-G depict root phenotypes of different genetic stocks with increasing pigmentation (from A to G) in the epidermis, outer phloem (cortex), inner phloem, and xylem tissues. (Modified from [19]); Supplementary Figure S2. Phenotype of the carrot roots and their root sections of samples used for transcriptome analysis; Supplementary Figure S3. Venn diagrams depicting the number of differentially expressed genes (DEGs) identified in the *P*_1_ (panel A), *P*_3_ (panel B), and *RIPAP* (panel C) regions of Chr. 3 for all the pairwise transcriptome comparisons between purple outer phloem (POP) and non-purple inner phloem (NPIP) in dark purple (dP1, dP2), and pale purple (pP) carrot roots; Supplementary FigureS4. Phylogenetic relationships among 22 carrot MYB transcription factors (indicated with red circles) and MYBs from other species known to be involved in the regulation of different flavonoid compounds; Supplementary Figure S5. Phylogenetic relationships among four carrot bHLH transcription factors (red circles) and bHLH genes from other species; Supplementary Figure S6. Phylogenetic relationships among six carrot ERF (indicated in red circles) and ERF genes from other species; Supplementary Figure S7. Phylogenetic relationships among two carrot NAC transcription factors (red circles) and NAC genes from other species with known biological functions; Supplementary Figure S8. Phylogenetic relationships among 12 carrot WRKY transcription factors (red circles) and WRKY genes from other species with known functions; Supplementary Figure S9. Phylogenetic relationships among MADS-box transcription factors from diverse plant species, including the carrot MADS-box gene DCAR_010757 colocalized with *ROPAP* and *RIPAP* in the *P*_3_ region (red dot). Supplementary Table S1. Characteristics of the plant materials and sequencing libraries used for transcriptome (RNA-seq) analysis, and number of reads obtained for each sample; Supplementary Table S2. Normalization factors for pair-wise transcriptome comparisons; Supplementary Table S3. Genes and primer sequences used for RT-qPCR analysis; Supplementary Table S4. Differentially expressed genes (DEGs) detected in pairwise transcriptome (RNA-Seq) comparisons of ‘purple outer phloem (POP)’ vs. ‘non-purple inner phloem (NPIP)’ in carrot roots varying in anthocyanin concentration and classified as dark purple (dP1, dP2), and pale purple (pP); Suplementary Table S5. Differentially expressed genes (DEGs) identified in the *P*_1_, *P*_3_, and *RIPAP* regions of Chr 3 for each pair-wise transcriptome comparisons performed between purple outer phloem (POP) and non purple inner phloem (NPIP) tissues from carrot roots varying in anthocyanin concentration and classified as dark purple (dP1, dP2), and pale purple (pP); Supplementary Table S6. Differentially expressed genes (DEGs) in the *P*_1_, *P*_3_, and *RIPAP* regions, consistently identidied in all the pairwise comparisons of purple outer phloem (POP) vs. non-purple inner phloem (NPIP); Supplementary Table S7. Transcription factors consistently upregulated (red) or downregulated (green) in the root purple outer phloem (POP) across all the pairwise transcriptome comparisons between POP and non-purple inner phloem (NPIP); Supplementary Table S8. Carrot DEGs belonging to the MYB, bHLH, Ethylene-responsive, NAC and WRKY transcription factor families consistenlty upregulated (in red) or downregulated (in green) in POP tissues across all the POP vs. NPIP transcriptome comparisons; Supplementary Table S9. MYB, bHLH, ERF, NAC and WRKY transcription factor gene sequences from different plant species used for phylogenetic analysis; Supplementary Table S10. Structural genes involved the flavonoid and anthocyanin biosynthetic pathways annotated in the carrot genome sequence; Supplementary Table S11. Summarized performance of 28 candidate genes for *ROPAP* and *RIPAP* across different gene expression and phylogenetic analyses performed in different carrot genetic backgrounds.
