# Supplementary material for: Genetic and Transcription Profile Analysis of Tissue-Specific Anthocyanin Pigmentation in Carrot Root Phloem
Source: Genes (Basel). 2021 Sep 22;12(10):1464. doi: 10.3390/genes12101464 (PMC8535259; doi:10.3390/genes12101464)
Supplement: Supplementary file 1 [file genes-12-01464-s001.zip › Supplementary Figures S1-S9_Revised - Bannoud et al..pdf]

SUPPLEMENTARY FIGURES S1-S9

List of figures in this file

Supplementary Figure S1. A. Cross-section of a carrot root from the 3242-F_2 mapping population, indicating the tissues that segregate for anthocyanin pigmentation in this background. **B-G.** Examples of the range of genetic variation in the carrot germplasm for anthocyanin pigmentation in different root tissues. Images B-G depict root phenotypes of different genetic stocks with increasing pigmentation (from A to G) in the epidermis, outer phloem (cortex), inner phloem, and xylem tissues. (Modified from [19]).

Supplementary Figure S2. Phenotype of the carrot roots and their root sections of samples used for transcriptome analysis. Sample sets with dark (dP1, dP2), and pale purple (pP) roots present high and low anthocyanin concentration in their outer phloem, respectively. Samples for RNA-seq were collected from taproot discs from the central portion of the root, from which purple outer phloem (POP) and non-purple inner phloem (NPIP) tissue samples were dissected and used for RNA isolation. Tissue samples were named with the sample set name followed by the tissue and color phenotype.

Supplementary Figure S3. Venn diagrams depicting the number of differentially expressed genes (DEGs) identified in the P_1 (panel A), P_3 (panel B), and *RIPAP* (panel C) regions of CHR 3 for all the pairwise transcriptome comparisons between purple outer phloem (POP) and non-purple inner phloem (NPIP) in dark purple (dP1, dP2), and pale purple (pP) carrot roots. DEGs in red and green were consistently upregulated and downregulated in POP, respectively.

Supplementary FigureS4. Phylogenetic relationships among 22 carrot *MYB* transcription factors (indicated with red circles) and *MYB*s from other species known to be involved in the regulation of different flavonoid compounds. The tree was constructed with MEGA X software using neighbor-joining phylogeny testing with 1,000 bootstrap replicates. Percentage values of replicate trees (>50) in which the associated taxa clustered together in the bootstrap test are indicated next to the branches. Accession numbers and gene IDs for the protein sequences used in the analysis are in Supplementary Table S9.

Supplementary Figure S5. Phylogenetic relationships among four carrot bHLH transcription factors (red circles) and bHLH genes from other species. The subgroups in the bHLH family were previously defined by [42,43]. Previously characterized bHLH genes from other species involved in the regulation of anthocyanin biosynthesis correspond to sub-group IIIf, indicated with an asterisk. The tree was constructed with MEGA X, using neighbor-joining phylogeny testing with 1,000 bootstrap replicates. Percentage values of replicate trees (>50) in which the associated taxa clustered together in the bootstrap test are indicated next to the branches. Accession numbers and gene IDs for the protein sequences used in the analysis are in Supplementary Table S9.

Supplementary Figure S6. Phylogenetic relationships among six carrot ERF (indicated in red circles) and ERF genes from other species. Based on the number of *AP2/ERF* domains and sequence similarities, the APETALA2/ETHYLENE RESPONSE FACTOR *AP2/ERF* superfamily is sub-classified into three groups; the ERF, AP2, and RAV families [44]. Previously characterized *ERF* genes from other species known to be involved in the regulation of

anthocyanin biosynthesis are highlighted in purple boxes (*PyERF3* and *MdERF1B*). The tree was constructed with MEGA X software using neighbor-joining phylogeny testing with 1,000 bootstrap replicates. Percentage values of replicate trees (>50) in which the associated taxa clustered together in the bootstrap test are indicated next to the branches.

Supplementary Figure S7. Phylogenetic relationships among two carrot NAC transcription factors (red circles) and NAC genes from other species with known biological functions. Previously characterized NACs from other species known to be involved in the regulation of anthocyanin biosynthesis are highlighted in purple box (*PpNAC1*). The tree was constructed with MEGA X software using neighbor-joining phylogeny testing with 1,000 bootstrap replicates. Percentage values of replicate trees (>50) in which the associated taxa clustered together in the bootstrap test are indicated next to the branches. Accession numbers and gene IDs for the protein sequences used in the analysis are in Supplementary Table S9.

Supplementary Figure S8. Phylogenetic relationships among 12 carrot WRKY transcription factors (red circles) and WRKY genes from other species with known functions. Clusters grouping were described by [45]. Previously characterized WRKY genes from other species known to be involved in the regulation of anthocyanin biosynthesis are highlighted in purple boxes (*PhPH3*, *AtTTG2*, *VvWRKY26* and *BnTTG2*). The tree was constructed with MEGA X software using neighbor-joining phylogeny testing with 1,000 bootstrap replicates. Percentage values of replicate trees (>50) in which the associated taxa clustered together in the bootstrap test are indicated next to the branches. Accession numbers and gene IDs for the protein sequences used in the analysis are in Supplementary Table S9.

Supplementary Figure S9. Phylogenetic relationships among MADS-box transcription factors from diverse plant species, including the carrot MADS-box gene DCAR_010757 colocalized with *ROPAP* and *RIPAP* in the P_3 region (red dot). MADS-box genes known to be involved in the regulation of anthocyanin biosynthesis are indicated with yellow stars. The tree was constructed using MEGA X, neighbor-joining phylogeny testing, with 1,000 bootstrap replicates. Percentage values of replicate trees in which the associated taxa clustered together in the bootstrap test are indicated next to the branches.

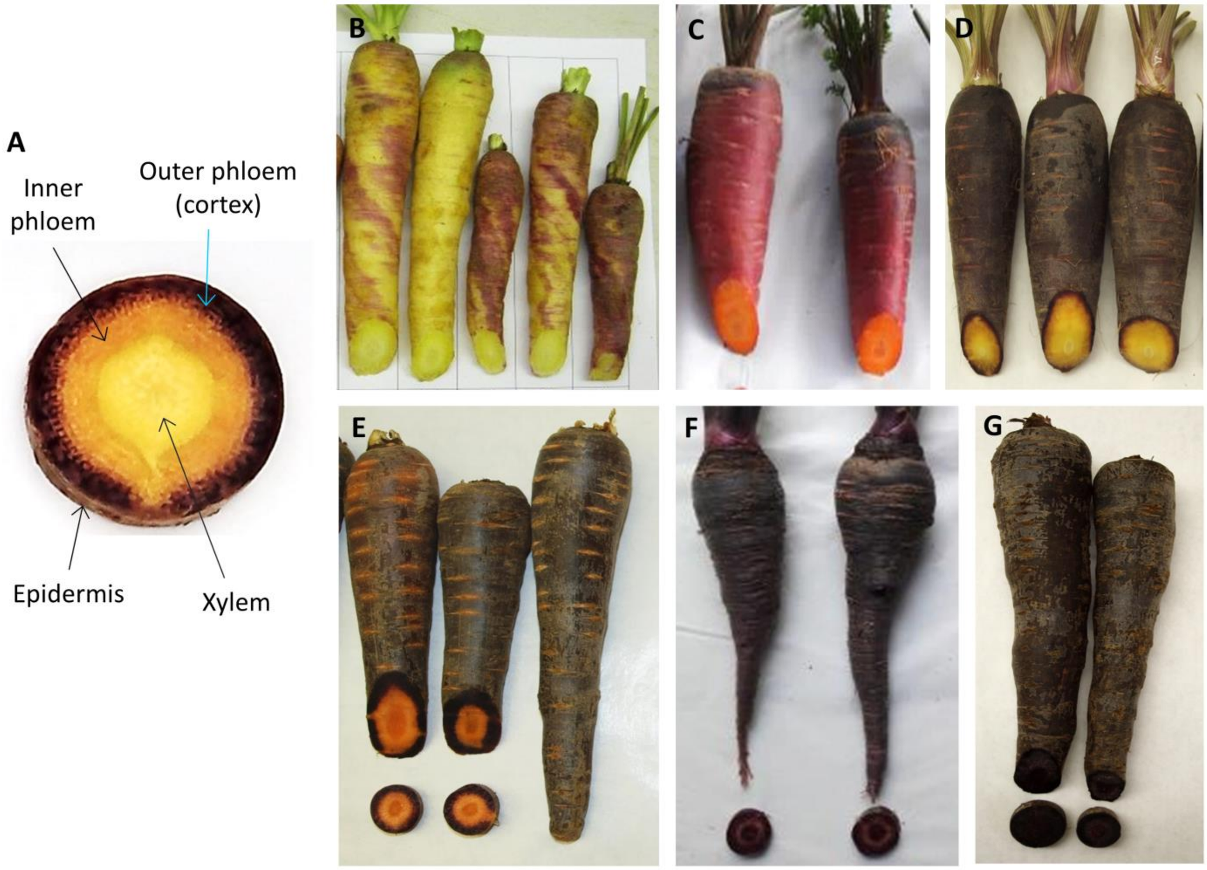

Supplementary Figure S1. A. Cross-section of a carrot root from the 3242-F_2 mapping population, indicating the tissues that segregate for anthocyanin pigmentation in this background. **B-G.** Examples of the range of genetic variation in the carrot germplasm for anthocyanin pigmentation in different root tissues. Images B-G depict root phenotypes of different genetic stocks with increasing pigmentation (from A to G) in the epidermis, outer phloem (cortex), inner phloem, and xylem tissues. (Modified from [19]).

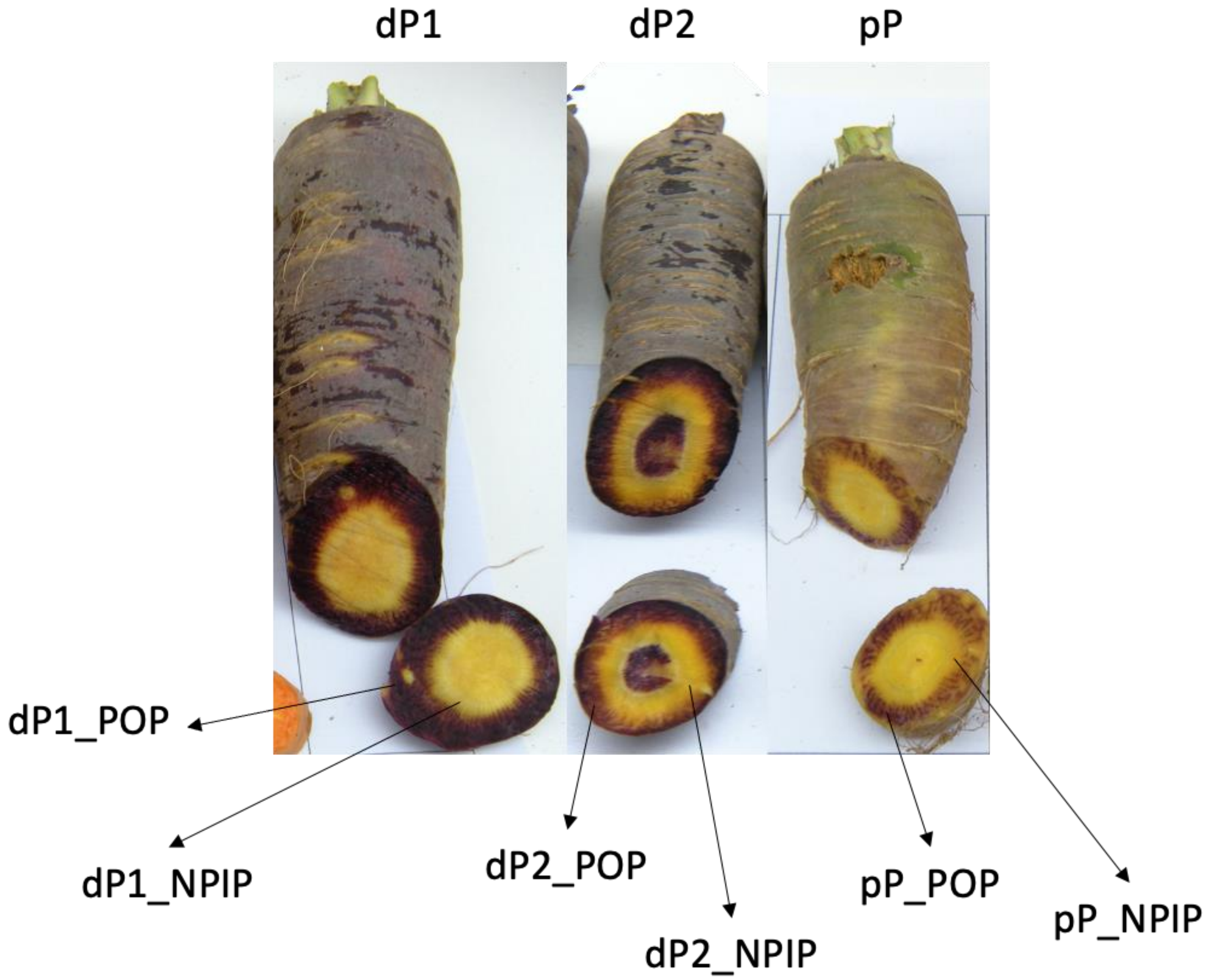

Supplementary Figure S2. Phenotype of the carrot roots and root sections of samples used for transcriptome analysis. Sample sets with dark (dP1, dP2), and pale purple (pP) roots present high and low anthocyanin concentration in their outer phloem, respectively. Samples for RNA-seq were collected from taproot discs from the central portion of the root, from which purple outer phloem (POP) and non-purple inner phloem (NPIP) tissue samples were dissected and used for RNA isolation. Tissue samples were named with the sample set name followed by the tissue and color phenotype.

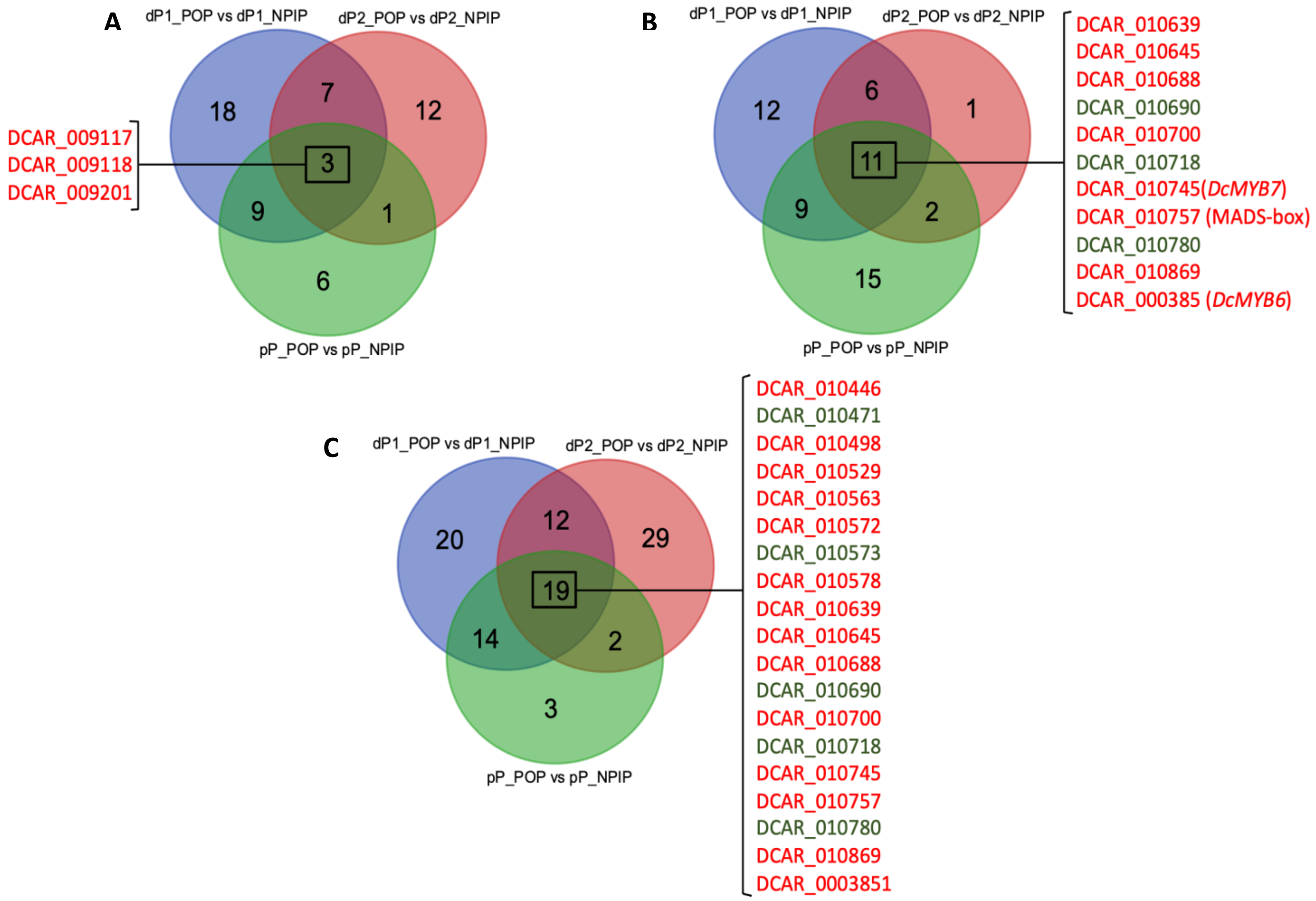

Supplementary Figure S3. Venn diagrams depicting the number of differentially expressed genes (DEGs) identified in the P_1 (panel A), P_3 (panel B), and *RIPAP* (panel C) regions of CHR 3 for all the pairwise transcriptome comparisons between purple outer phloem (POP) and non-purple inner phloem (NPIP) in dark purple (dP1, dP2), and pale purple (pP) carrot roots. DEGs in red and green were consistently upregulated and downregulated in POP, respectively.

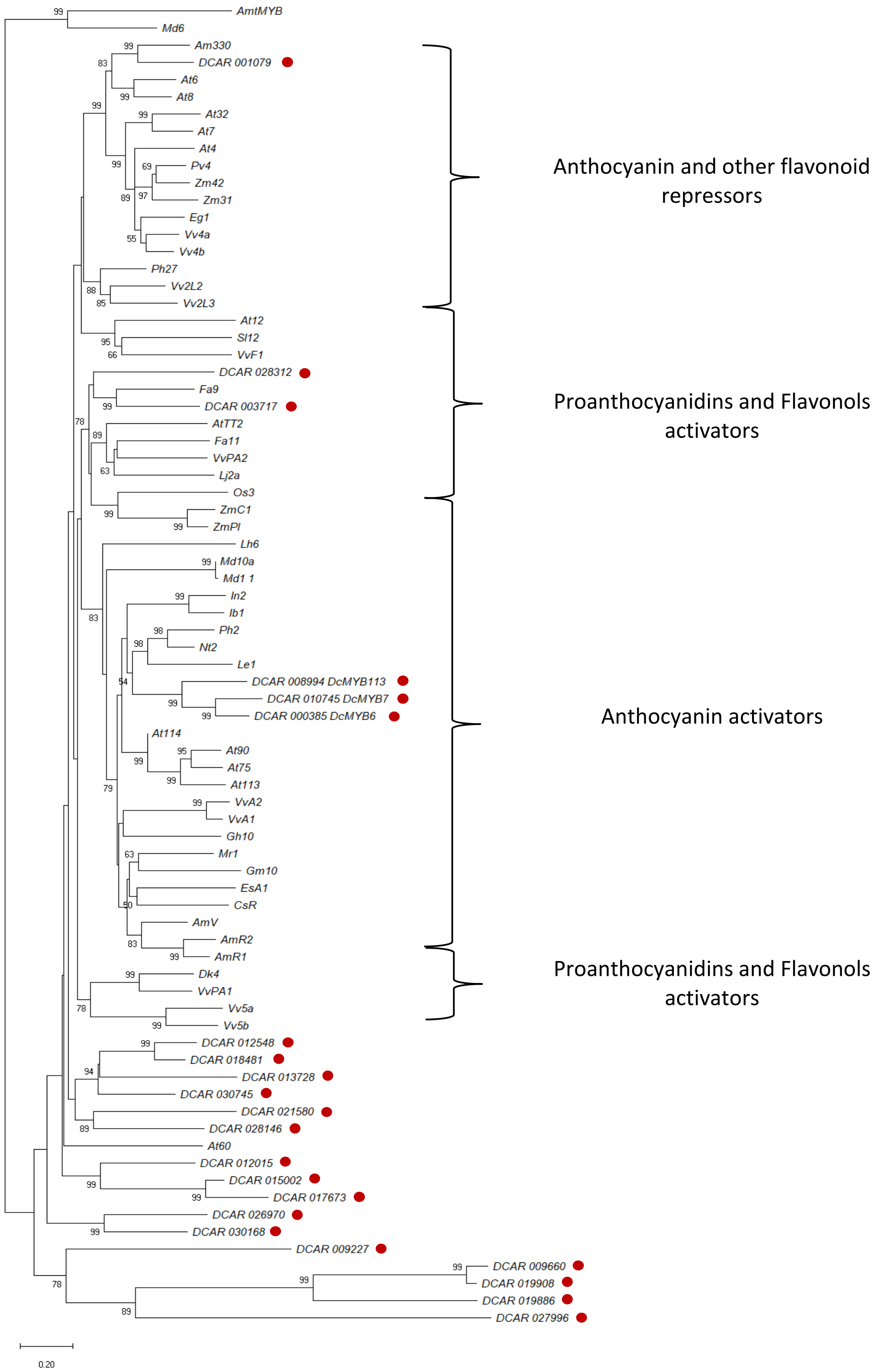

Supplementary Figure S4. Phylogenetic relationships among 22 carrot MYB transcription factors (indicated with red circles) and MYBs from other species known to be involved in the regulation of different flavonoid compounds. The tree was constructed with MEGA X software using neighbor-joining phylogeny testing with 1,000 bootstrap replicates. Percentage values of replicate trees (>50) in which the associated taxa clustered together in the bootstrap test are indicated next to the branches. Accession numbers and gene IDs for the protein sequences used in the analysis are in Supplementary Table S9.

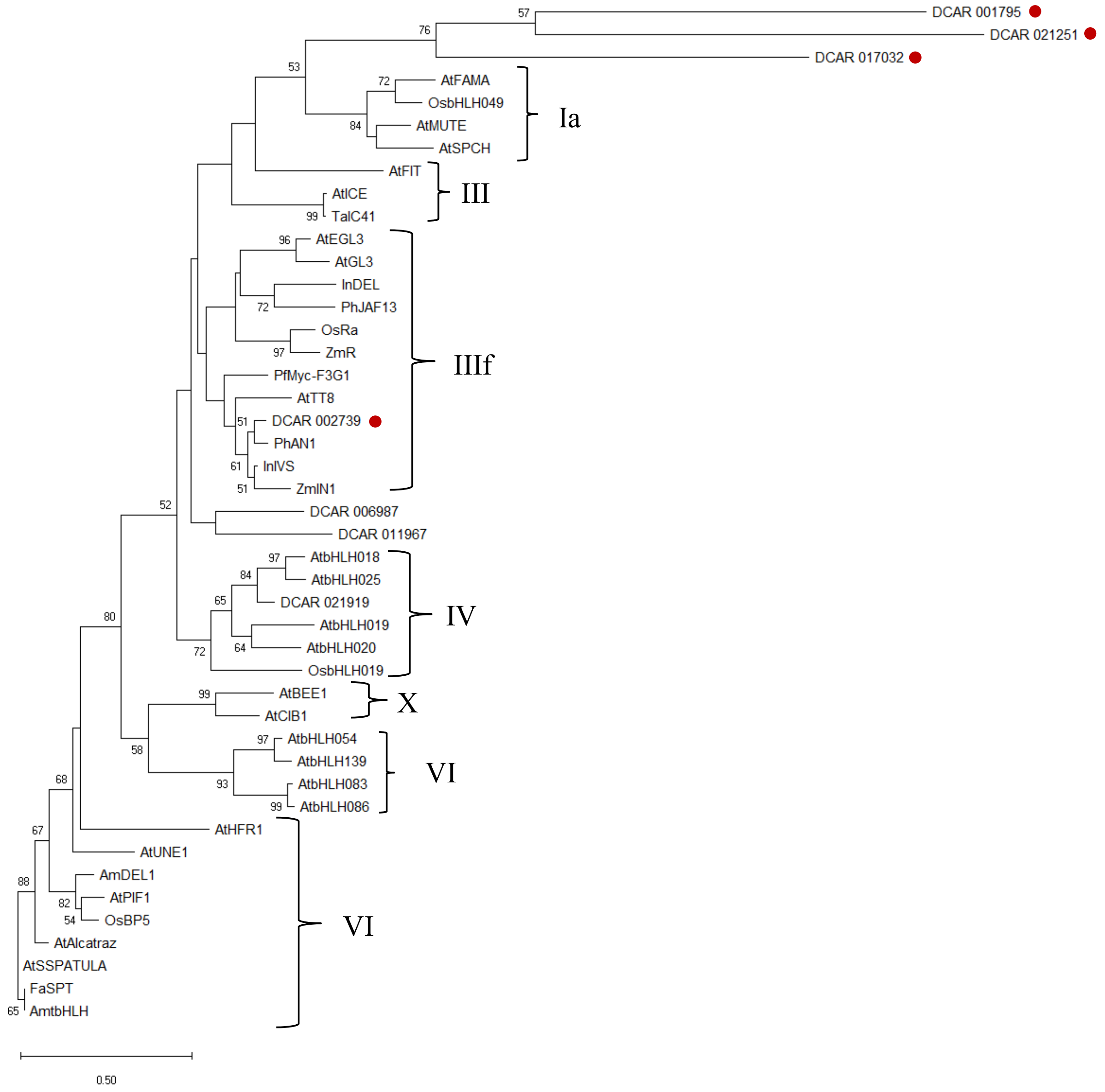

Supplementary Figure S5. Phylogenetic relationships among four carrot bHLH transcription factors (red circles) and bHLH genes from other species. The subgroups in the bHLH family were previously defined by [42,43]. Previously characterized bHLH genes from other species involved in the regulation of anthocyanin biosynthesis correspond to sub-group IIIf, indicated with an asterisk. The tree was constructed with MEGA X, using neighbor-joining phylogeny testing with 1,000 bootstrap replicates. Percentage values of replicate trees (>50) in which the associated taxa clustered together in the bootstrap test are indicated next to the branches. Accession numbers and gene IDs for the protein sequences used in the analysis are in Supplementary Table S9.

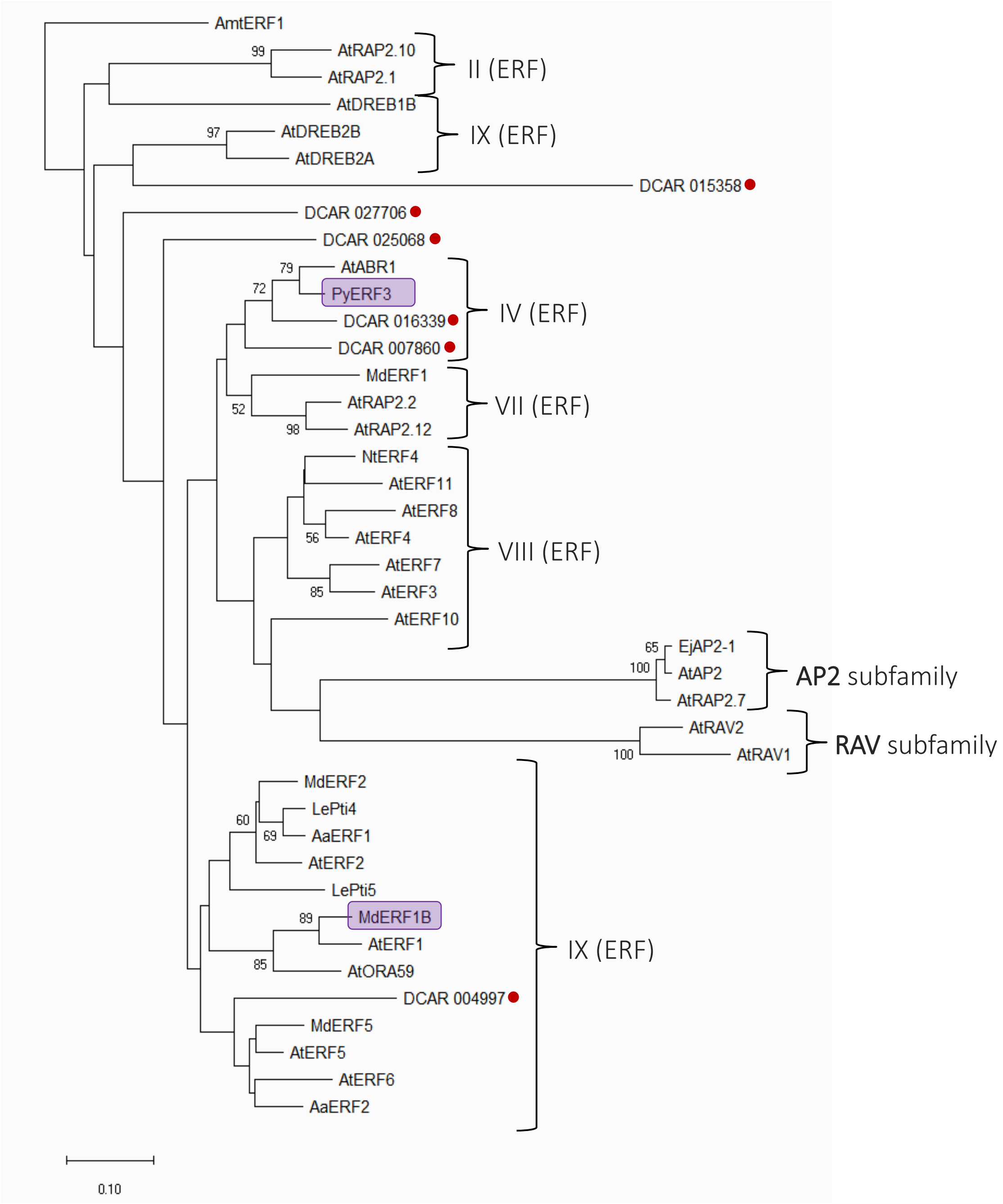

Supplementary Figure S6. Phylogenetic relationships among six carrot ERF (indicated in red circles) and ERF genes from other species. Based on the number of AP2/ERF domains and sequence similarities, the APETALA2/ETHYLENE RESPONSE FACTOR AP2/ERF superfamily is sub-classified into three groups; the ERF, AP2, and RAV families [44]. Previously characterized ERF genes from other species known to be involved in the regulation of anthocyanin biosynthesis are highlighted in purple boxes (*PyERF3* and *MdERF1B*). The tree was constructed with MEGA X software using neighbor-joining phylogeny testing with 1,000

bootstrap replicates. Percentage values of replicate trees (>50) in which the associated taxa clustered together in the bootstrap test are indicated next to the branches.

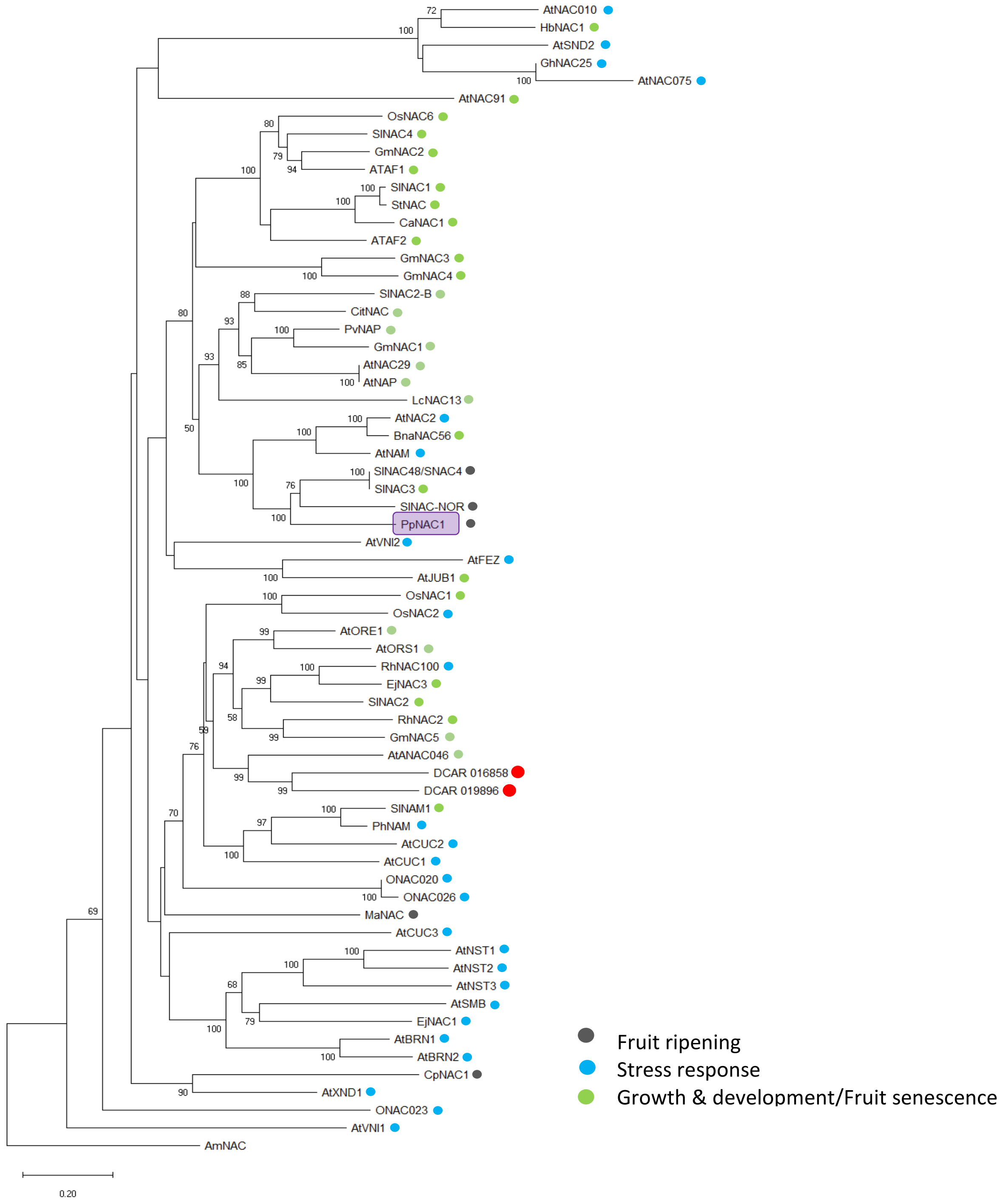

Supplementary Figure S7. Phylogenetic relationships among two carrot NAC transcription factors (red circles) and NAC genes from other species with known biological functions. Previously characterized NACs from other species known to be involved in the regulation of anthocyanin biosynthesis are highlighted in purple box (*PpNAC1*). The tree was constructed with MEGA X software using neighbor-joining phylogeny testing with 1,000 bootstrap replicates. Percentage values of replicate trees (>50) in which the associated taxa

clustered together in the bootstrap test are indicated next to the branches. Accession numbers and gene IDs for the protein sequences used in the analysis are in Supplementary Table S9.

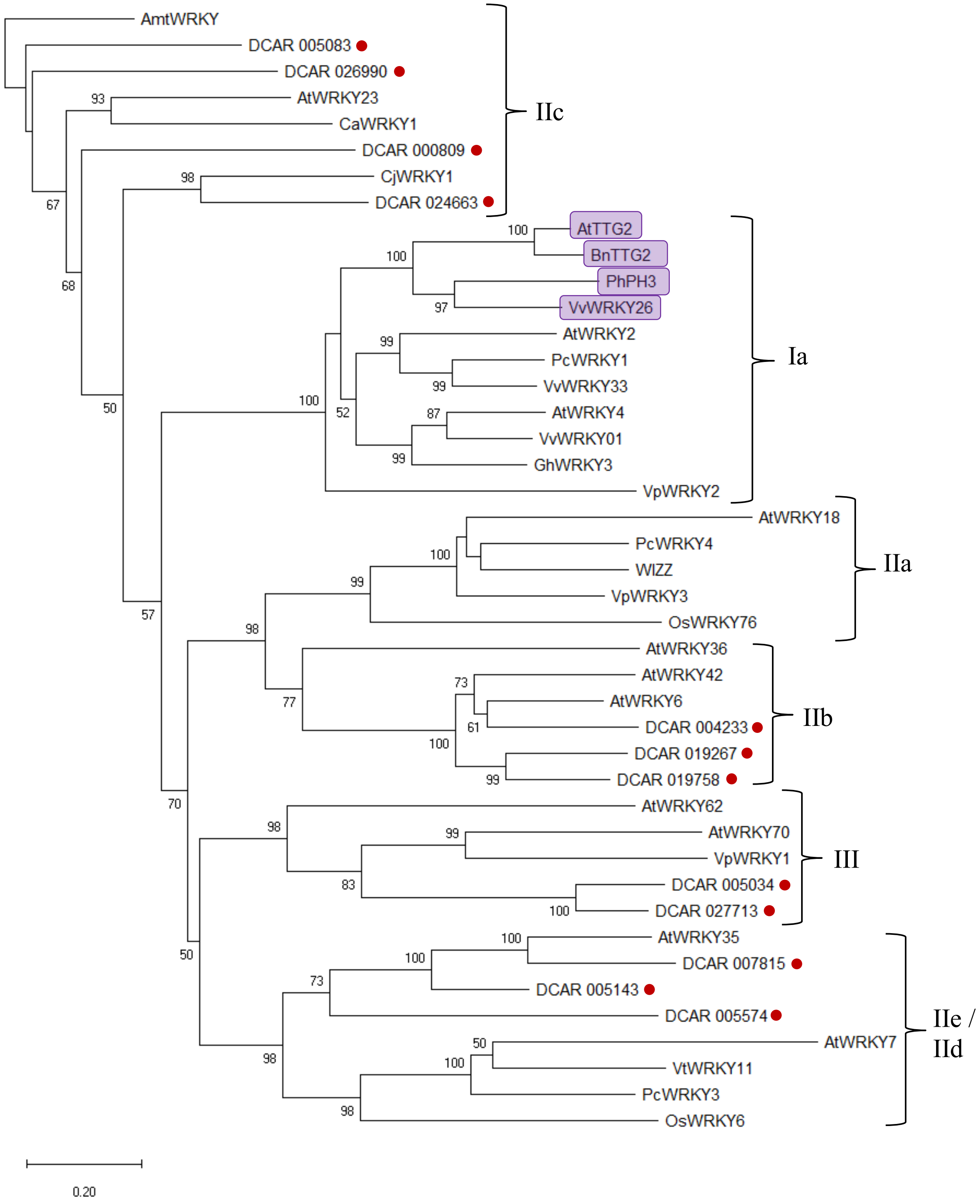

Supplementary Figure S8. Phylogenetic relationships among 12 carrot WRKY transcription factors (red circles) and WRKY genes from other species with known functions. Clusters grouping were described by [45]. Previously characterized WRKY genes from other species known to be involved in the regulation of anthocyanin biosynthesis are highlighted in purple boxes (*PhPH3*, *AtTTG2*, *VvWRKY26* and *BnTTG2*). The tree was constructed with MEGA X software using neighbor-joining phylogeny testing with 1,000 bootstrap replicates. Percentage values of replicate trees (>50) in which the associated taxa clustered together in the bootstrap test are indicated next to the branches. Accession numbers and gene IDs for the protein sequences used in the analysis are in Supplementary Table S9.

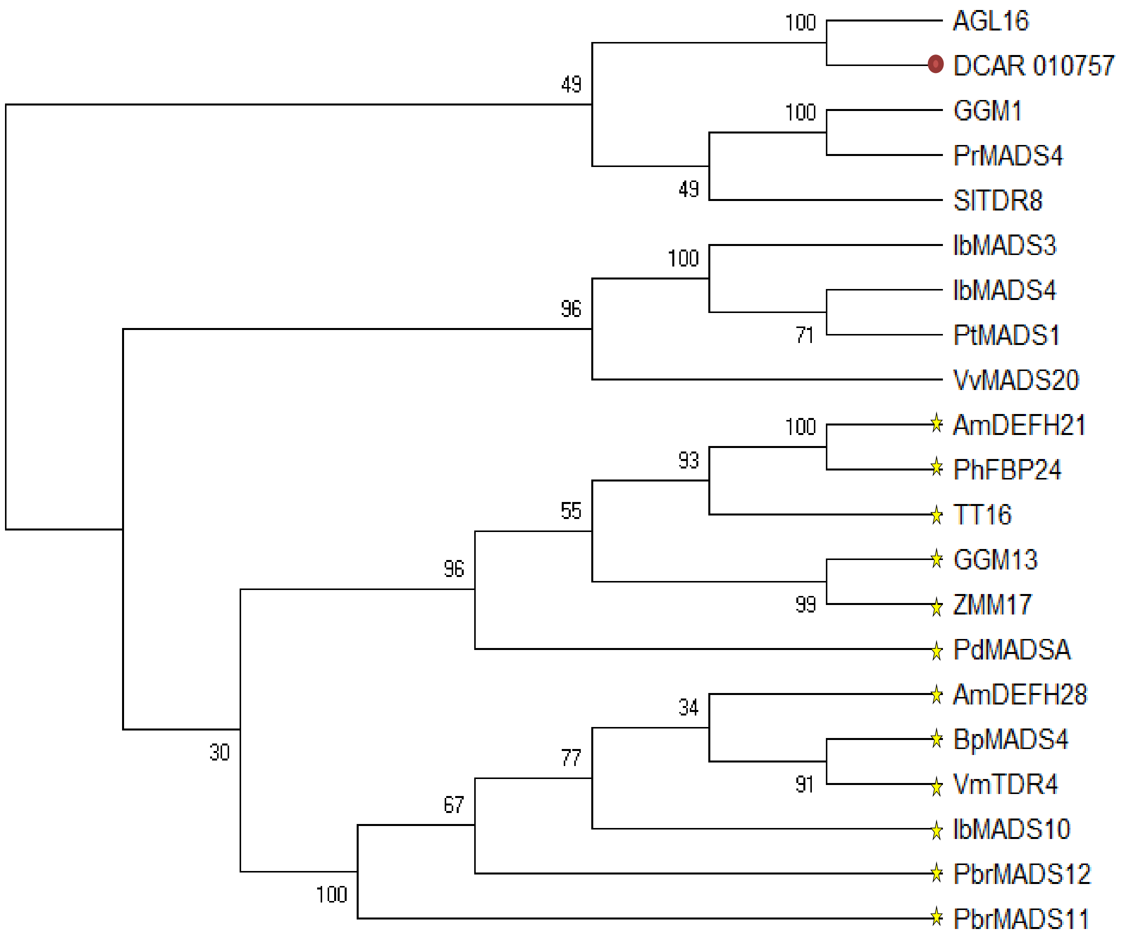

Supplementary Figure S9. Phylogenetic relationships among MADS-box transcription factors from diverse plant species, including the carrot MADS-box gene DCAR_010757 colocalized with *ROPAP* and *RIPAP* in the P_3 region (red dot). MADS-box genes known to be involved in the regulation of anthocyanin biosynthesis are indicated with yellow stars. The tree was constructed using MEGA X, neighbor-joining phylogeny testing, with 1,000 bootstrap replicates. Percentage values of replicate trees in which the associated taxa clustered together in the bootstrap test are indicated next to the branches.
